# Immuno-profiling of *Brucella* proteins for developing improved vaccines and DIVA capable serodiagnostic assays for brucellosis

**DOI:** 10.3389/fmicb.2023.1253349

**Published:** 2023-10-04

**Authors:** Prachita Nandini, Padmaja Jakka, Subathra Murugan, Varadendra Mazumdar, Deepak Kumar, Richa Prakash, Sukhadeo B. Barbuddhe, Girish Radhakrishnan

**Affiliations:** ^1^National Institute of Animal Biotechnology (NIAB), Hyderabad, India; ^2^Regional Centre for Biotechnology (RCB), Faridabad, India; ^3^ICAR- National Meat Research Institute, Hyderabad, India

**Keywords:** *Brucella*, vaccine, DIVA, immunoprofiling, ELISA, serodominant, diagnostics

## Abstract

Brucellosis remains a worldwide zoonotic disease with a serious impact on public health and livestock productivity. Controlling brucellosis in livestock is crucial for limiting human infections in the absence of effective human vaccines. Brucellosis control measures are majorly dependent on rigorous monitoring of disease outbreaks and mass vaccination of livestock. Live attenuated vaccines are available for livestock vaccination that play a vital role in brucellosis control programs in many countries. Even though the existing animal vaccines confer protection against brucellosis, they carry some drawbacks, including their infectivity to humans and interference with sero-monitoring. The available serodiagnostic assays for brucellosis depend on detecting anti-LPS antibodies in the serum. Since diagnosis plays a vital role in controlling brucellosis, developing improved serodiagnostic assays with enhanced specificity, sensitivity and DIVA capability is required. Therefore, it is essential to identify novel antigens for developing improved vaccines and serodiagnostic assays for brucellosis. In the present study, we performed a high throughput immunoprofiling of *B. melitensis* protein microarray using brucellosis-positive human and animal serum samples. The screening identified several serodominant proteins of *Brucella* that exhibited common or differential reactivity with sera from animals and humans. Subsequently, we cloned, expressed, and purified ten serodominant proteins, followed by analyzing their potential to develop next-generation vaccines and improved serodiagnostic assays for brucellosis. Further, we demonstrated the protective efficacy of one of the serodominant proteins against the *B. melitensis* challenge in mice. We found that the seroreactive protein, Dps (BMEI1980), strongly reacted with brucellosis-positive serum samples, but it did not react with sera from *B. abortus* S19-vaccinated cattle, indicating DIVA capability. A prototype lateral flow assay and indirect ELISA based on Dps protein exhibited high sensitivity, specificity, and DIVA capability. Thus, the present study identified promising candidates for developing improved vaccines and affordable, DIVA-capable serodiagnostic assays for animal and human brucellosis.

## Introduction

Brucellosis is one of the major economically important zoonotic diseases worldwide, which is posing a serious threat to both livestock and human health globally (Mcdermott et al., 2013). Besides its impact on economic loss, brucellosis is also associated with high morbidity in many developing countries, both in humans and animals (Rubach et al., 2013). Brucellosis remains endemic in different regions, including Latin America, the Middle East, Africa, Asia, and the Mediterranean basin (Pappas et al., 2006). Brucellosis is caused by the Gram-negative facultative intracellular bacteria, *Brucella* belonging to the class Alphaproteobacteria (Ficht, 2010). Depending on the host preference and pathogenicity, 12 species of *Brucella* have been identified to date, which can infect domestic, wild, and marine animals (Scholz et al., 2016). Four classical *Brucella* species capable of causing severe brucellosis in humans are *B. melitensis*, followed by *B. abortus*, *B. suis*, and *B. canis*. Symptoms of brucellosis in animals are abortions, death of weaker offspring, stillbirth, and sterility in males, while in humans, it mainly causes flu-like symptoms with high fever, headache, weakness, and osteoarticular problems (Godfroid et al., 2011). A severe form of brucellosis could be fatal with cardiac and neurological complications (Solera et al., 1997). Brucellosis in humans is often an occupational hazard directly affecting veterinarians, laboratory technicians, abattoir workers, and farmers (Fiori et al., 2000; Agasthya et al., 2007). Human transmission could mainly occur through direct contact with infected or aborted animals, consuming contaminated dairy products, and inhaling aerosols (Corbel, 1997). There is no human vaccine for brucellosis, and treatment with multiple antibiotic regimens is the only option to treat human brucellosis. However, antibiotic treatment is challenging due to frequent therapeutic failures and relapses (Ariza et al., 2007).

The most plausible way to control brucellosis is early disease diagnosis and mass vaccination of susceptible animals. However, diagnosis of brucellosis is challenging, and the available animal vaccines for brucellosis have many disadvantages despite conferring protection (Schurig et al., 2002; Galińska and Zagórski, 2013). Brucellosis exhibits non-specific clinical manifestations that mimic the symptoms of other infectious or non-infectious diseases (Mantur et al., 2007). Direct culturing of *Brucella* from the tissue specimens is considered the gold standard for diagnosing brucellosis. However, it is often avoided due to its fastidious nature, slow growth, and potential hazard to laboratory personnel (Padilla Poester et al., 2010). Therefore, other diagnostic methods have been developed and practiced over time that require no direct contact and do not require expertise and special equipment (Nielsen and Yu, 2010). Therefore, serological detection of brucellosis is one of the most preferred diagnostic tools since this method addressed most of the challenges stated earlier. The serodiagnosis of brucellosis mainly relies on detecting anti-lipopolysaccharide (LPS) antibodies in the infected serum (Godfroid et al., 2010). However, LPS-based serodiagnostic assays have significant drawbacks, including poor sensitivity, cross-reactivity with several other Gram-negative bacteria, and lack of the capability of Differentiating Infected from Vaccinated Animals (DIVA). Also, the tests fail to detect rough strains of *Brucella*, *viz. B. canis* and *B. ovis*, which lack O-side chains on their LPS (Al Dahouk et al., 2003a,b). Hence it is crucial to identify other serodominant antigens of *Brucella* that are exclusive and capable of addressing major shortcomings of the present serodiagnostic assays.

Among the available animal vaccines, S19 and RB51 are the well-recognized live attenuated vaccines used for cattle (Avila-Calderón et al., 2013). However, there are many disadvantages associated with these vaccines. The immune response induced by the S19 vaccine is still unclear, and it can induce abortion in pregnant animals. *B. abortus* S19 vaccine has no DIVA capability, and most importantly, it is highly infectious to humans (Yang et al., 2013). RB51, a rough live attenuated strain of *B. abortus*, has DIVA capability where the vaccinated cattle can be differentiated from naturally infected animals using routine serodiagnostic tests (Ashford et al., 2004). However, RB51 is rifampicin-resistant and can cause infection in humans. Subunit vaccines using *Brucella* recombinant proteins such as L7/L12 ribosomal protein, Cu-Zn superoxide dismutase, and Outer Membrane Lipoproteins (OMPs) OMP16 and OMP19 have been tested for brucellosis (Oliveira and Splitter, 1996; Bae, 1999; Pasquevich et al., 2009; Sáez et al., 2012). However, no subunit vaccines are currently available to prevent brucellosis in animals and humans. Therefore, the identification and characterization of novel serodominant antigens of *Brucella* are needed for developing improved vaccines and diagnostic assays for brucellosis. Here, we performed a high throughput immunoprofiling of *B. melitensis* antigens using a protein microarray. The screening identified several serodominant proteins of *Brucella* that are uniquely present or shared by various host species. Subsequently, we performed a detailed characterization of 10 serodominant proteins to assess their utility for developing improved vaccines and serodiagnostic assays. Further, we developed a prototype Lateral Flow Assay and indirect ELISA based on one of the serodominant proteins. The assays could detect brucellosis in humans and animals with high sensitivity and specificity and exhibited DIVA capability.

## Materials and methods

### Ethical statement

Six to eight-week-old female BALB/c mice were procured from the Small Animal Facility of the National Institute of Animal Biotechnology (NIAB). All mice experiments and animal serum collections were approved by the Institutional Biosafety Committee (Approval number: IBSC/2013/NIAB/0001B) and Institutional Animal Ethics Committee (Approval number: IAEC/2019/NIAB/34/GKR & IAEC/2021/NIAB/09/GKR) of NIAB. *In vivo* challenge studies with *B. melitensis* 16 M strain were performed in the BSL-3 laboratory facility of UoH-NIAB (Approval number: IAEC/NIAB/2022/08/GKR). Mice were kept under a standard pathogen-free environment and handled with humane care with free access to food and water throughout the experiment. Serum samples from human subjects were collected by ICAR-National Meat Research Institute with approval from the Institutional Ethics Committee (Approval number: KAMSRC/IEC/04/2018).

### High-throughput immunoprobing of the *Brucella melitensis* protein microarray

*Brucella melitensis*, Bv.1 strain 16 M proteome microarrays with a coverage of 99.5% were commercially procured from Antigen Discovery, USA (Cat. No. 12-MA-0001). The nitrocellulose microarray slides printed with ~3,000 *Brucella* proteins were analyzed with healthy and brucellosis-positive serum samples. Healthy and brucellosis-positive serum samples were obtained from veterinary diagnostic centers and farms with necessary approvals (Approval number: IBSC/2013/NIAB/0001B). *B. abortus* S19-vaccinated serum samples were obtained from different villages through government agencies as part of the ongoing brucellosis control program in India. Serum samples were primarily screened for the presence of seropositivity using the Rose Bengal Plate Agglutination Test (RBPT) and the presence of anti-*Brucella* antibodies was confirmed using OIE-validated, PrioCHECK^™^ Brucella Ab 2.0 Strip Kit (Applied Biosystems) and IDEXX Brucellosis Serum X2 Ab Test multi-species ELISA for animal sera. NovaLisa^®^ human IgG and NovaLisa^®^ human IgM kits (Nova Tec, Germany) for screening human sera. To perform RBPT, 25 μL of Rose Bengal *Brucella* antigen and 25 μL of serum sample were added onto the glass slides and mixed, followed by incubation for 5 min and observation for agglutination. Indirect ELISAs were performed with the collected serum samples as per the manufacturer’s protocols.

Immunoprobing of protein microarray was performed as per instructions of the manufacturer. Briefly, the printed slides were assembled and blocked with 350 μL of blocking buffer for 1 h at room temperature with gentle agitation. The kit control or test serum samples were diluted at a ratio of 1:100 in the pre-incubation solution (20% *E. coli* lysate in the blocking buffer) and incubated for 30 min before adding onto the microarray slides. Subsequently, 350 μL of diluted sera were added and incubated overnight at 4° C in a humidified airtight container. Next, the slides were washed three times with 1 mL of wash buffer for 5 min each, followed by the addition of 350 μL of diluted secondary antibody (1:1,000) onto the slides and incubated for 1 h at room temperature. The slides were then washed thrice, followed by the addition of 350 μL of diluted tertiary detection antibody and incubated for 1 h at room temperature in the dark. The slides were given a final wash and air-dried before being scanned with the Axon Genepix scanner (Molecular Devices).

The background (true negative) signal was defined as the average signal of the negative control spots on the array. This enabled the comparison of the individual protein signal with the background signal to determine the significant response by protein antigens. The average of replicated spots was measured per sera, and the signal intensities obtained for sera from individual species were analyzed using the Genepix Pro 6.0 software.

### Cloning and expression of serodominant proteins of *Brucella*

The genes encoding the identified serodominant protein antigens were amplified from the chromosomal DNA of *B. melitensis* using respective forward and reverse primers harboring *BamH1* and *Xho1* enzymes (Table 1). The PCR amplicons were digested with restriction enzymes and gel eluted. The purified PCR products were cloned into *BamH1* and *Xho1* sites of pET21a(+) in-frame with a 6X Histidine (His) tag at the C-terminus. Subsequently, the clones were confirmed by restriction enzyme digestion and sequencing. The pET21a(+) plasmids harboring the cloned *Brucella* genes were then introduced into BL21 (DE3) *E. coli*. To examine the overexpression of *Brucella* proteins, BL21 cells were induced with 1.0 mM Isopropyl β-D-1-thiogalactopyranoside (IPTG) for 5 h at 37°C, followed by harvesting the cells. The cells were lysed in 2X SDS samples buffer, boiled for 10 min, and analyzed by SDS-PAGE.

**Table 1:** Primers used for amplification of open reading frames of 10 serodominant proteins.

Gene ID	Primer sequence
BMEI1980	F-5′CGCGGATCCGTGCGATCGCCATTT-3′ R-5′CCGCTCGAGATTGCTTTCCTGCACA-3′
BMEI1390	F-5′CGCGGATCCATGACGGCGGGCGC-3′ R-5′CCGCTCGAGGAATGGAGAATCTGGGA-3′
BMEI1513	F-5′CGCGGATCCATGCGCGATCCCTAT-3′ R-5′CCGCTCGAGCACAACCCTGCGTTT-3′
BMEI0063	F-5′CGCGGATCCGTGGGGCAGGGG-3′ R-5′CCGCTCGAGTGTAAAATTAAAGTTTC-3′
BMEI0856	F-5′CGCGGATCCATGCCGATCAATATCACC-3′ R-5′CCGCTCGAGGACCAGCATACCCATC-3′
BMEI0916	F-5′CGCGGATCCATGCGCGACGGCGTA-3′ R-5′CCGCTCGAGGTCGACAATGTCATCG-3′
BMEII1048	F-5′CGCGGATCCATGGCTGCAAAAGAC-3′ R-5′CCGCTCGAGGAAGTCCATGCCGCC-3′
BMEI0855	F-5′ATTTGCGGCCGCATGCCCATAGAAAT-3′ R-5′CCGCTCGAGAGCGGTATAGGTAACG-3′
BMEII0154	F-5′ATTTGCGGCCGCATGAACATTGAG-3′ R-5′CCGCTCGAGTGGCTTGGACTTGAT-3′
BMEI0748	F-5′CGCGGATCCATGGCTGATCTCGCA-3′ R-5′CCGCTCGAGCTTGAGTTCAACCTTGG-3′

### Purification of His-tagged serodominant proteins of *Brucella*

The BL21 (DE3) *E. coli* harboring individual *Brucella* protein expression plasmids were grown overnight at 37°C in Lauria Bertini media (LB, Himedia) supplemented with ampicillin (100 μg/mL; Sigma). This starter culture was inoculated into 1 liter of LB broth in 1/100 dilution and incubated at 37°C until the OD at 600 nm reached 0.6. The bacterial cultures were then induced with 1.0 mM IPTG and incubated further for 5 h at 37°C. Subsequently, the bacterial cells were harvested by centrifugation at 6,000 × *g* at 4°C and washed once with PBS. Next, the bacterial cells were lysed in 50 mL of sonication buffer containing 50 mM sodium phosphate buffer with 300 mM NaCl and 10 mM imidazole. The cell lysates were clarified by centrifugation at 16,000 × *g* for 20 min at 4°C, followed by the supernatant collection. Subsequently, the supernatant was passed through the pre-equilibrated column packed with 1 mL of Nickel-NTA Agarose resin (QIAGEN) at a flow rate of 1 mL/min. The Ni-NTA column was then washed with 200 mL of ice-cold wash buffer containing 50 mM sodium phosphate buffer with 300 mM NaCl and 30 mM imidazole. The His-tagged protein immobilized on the Ni-NTA resin, was then eluted with the same buffer containing 300 mM imidazole in various fractions. Next, SDS-PAGE was performed with the eluted fractions to assess their purity, and the protein-containing fractions were pooled. The residual LPS from the purified proteins was removed using polymyxin-B immobilized columns (Thermo Scientific) as per the manufacturer’s protocol. Subsequently, the purified proteins were dialyzed against PBS overnight. After estimating the concentration by Bradford assay (Sigma), the proteins were aliquoted, followed by snap freezing and storing at −80°C for further experiments.

### Immunoblot analysis of purified proteins

The protein samples were mixed with 2X Laemmli buffer, followed by boiling for 10 min at 100°C. Next, the samples were resolved on 12% SDS-PAGE gel, followed by the transfer of proteins onto PVDF membranes using a wet tank blotting system (Bio-Rad). The membrane was blocked with 5% skimmed milk in TBST (Cell Signaling Technology) for 1 h, followed by incubation with HRP-conjugated anti-His-tag antibody (R&D Systems) at 1:10,000 dilution overnight at 4°C. Subsequently, the membrane was washed 3 times with TBST and incubated with Super Signal West Pico or Femto chemiluminescent substrate (Pierce). The signals were captured using a chemi-documentation system (Syngene).

To analyze the seroreactivity, the purified recombinant proteins were transferred onto the PVDF membrane as described above. Next, the membranes were blocked with 5% skimmed milk in TBST, followed by incubating with brucellosis positive or negative sera (1:100 dilution) from cattle overnight at 4°C. Next, the membranes were washed 3 times with TBST and incubated with HRP-conjugated anti-bovine IgG at 1:5,000 dilution (ThermoFisher). Subsequently, the membranes were incubated with the chemiluminescent substrate (Pierce), and the signals were captured using a chemi-documentation system (Syngene).

To examine the seroreactivity of Dps protein with brucellosis-positive human and animal sera, Dps protein was resolved on denaturing or native SDS-PAGE gel, followed by immunoblotting. The membranes were blocked with 5% skimmed milk in TBST and incubated with brucellosis-positive human and animal sera (1:100 dilution) overnight at 4°C. Next, the membranes were washed 3 times with TBST and incubated with HRP-conjugated anti-species specific IgG at 1:5,000 dilution (Thermo Fisher), followed by detection of the signal as described before.

To examine the differential recognition of purified recombinant Dps protein by *B. abortus* S19-vaccinated and naturally-infected cattle sera, increasing concentrations of Dps protein along with *B. abortus* and *E. coli* LPS were subjected to 12% SDS-PAGE, followed by immunoblotting as described above. The membranes were incubated with *B. abortus* S19-vaccinated or naturally infected cattle sera. Subsequently, the immunoblots were probed with HRP-conjugated anti-bovine IgG at 1:5,000 dilution (ThermoFisher), followed by detection of the signal as described before.

To assess the seroreactivity of Dps with S19-vaccinated cattle sera after various days post-vaccination, serum samples were collected at 21-, 45-, and 90-days post-vaccination, followed by probing the membrane harboring Dps protein as described before. To examine the expression of Dps protein, total lysates of *B. abortus* and *B. abortus* S19 were resolved by SDS-PAGE, followed by the transfer of the proteins onto the PVDF membrane. The membrane was probed with an anti-Dps antibody (1:5,000), which was generated commercially (GenScript) or serum from L7/L12 immunized mice, followed by detection of the signal as described before.

To examine the reactivity of *S. typhimurium* or *E. coli* lysates with anti-Dps antibody, 40 μg of whole cell lysate of *S. typhimurium, E. coli, B. abortus or* purified Dps protein (10 μg) was resolved on 12% SDS-PAGE gel, followed by transfer of proteins onto PVDF membrane. The membrane was probed with anti-Dps antibody at a dilution of 1:5000 to detect the Dps proteins as described before.

### Immunization of mice with purified serodominant proteins

Six to eight-week-old female BALB/c mice were used for analyzing the immunogenicity of purified proteins. Individual animal of each designated group (six mice per group) was intraperitoneally administrated with 40 μg of purified recombinant protein mixed with Freund’s complete adjuvant (1:1 ratio). Subsequently, the mice were given a booster dose of 20 μg of protein complexed with Freund’s incomplete adjuvant on day 21 (1:1 ratio). The mice injected with recombinant L7/L12 protein of *B. melitensis* were considered the positive control group, and the mice injected with adjuvant alone were considered the negative control group (Oliveira et al., 1994; Oliveira and Splitter, 1994, 1996; Singh et al., 2015). The blood was collected from individual mice from each group on 0, 14, 21, 28, 35, and 45 days after initial immunization through the retro-orbital route. On the 45th day, mice were euthanized, and spleens were removed aseptically to isolate the splenocytes for further immunological assays.

### Analyzing the humoral and cell-mediated immune responses in immunized mice

The antibody response against the purified proteins was estimated by iELISA (indirect enzyme-linked immunosorbent assay) in the serum samples collected at various time intervals. Initially, 96-well microtiter plates were coated overnight with respective purified recombinant proteins (100 ng/well) at 4°C. The wells were then washed thrice with 300 μL of 1X phosphate buffered saline—tween 20 (PBST) to remove the unbound proteins and blocked for 1 h at room temperature with 100 μL/ well of 1% BSA prepared in phosphate buffer saline (PBS). The wells were washed thrice with 300 μL of 1X PBST, followed by adding serum samples (100 μL/ well) diluted in 1% BSA at a ratio of 1:100 to the respective wells. The plates were incubated at room temperature for 2 h. Subsequently, the wells were washed again as described earlier, followed by incubation with 100 μL/ well of HRP-conjugated goat anti-mouse IgG1 or IgG2a antibody (Novus Biologicals) at a dilution of 1:10,000 for 1 h at 37° C. Next, the wells were washed and incubated with 50 μL of TMB substrate for 10–15 min, and the reaction was stopped by adding 50 μL of 1 N H_2_SO_4_. The absorbance of the wells was measured at 450 nm using a microplate reader (PerkinElmer).

To analyze the population of CD4^+^, CD8^+^, and IFN-γ producing CD4^+^ and CD8^+^ T cells, the spleens were aseptically harvested from immunized mice, followed by homogenization in sterile PBS. Subsequently, a single-cell suspension was prepared by passing the homogenized samples through 70 μm cell strainers (BD Biosciences). The cells were washed twice with 10 mL of PBS, and residual RBCs were lysed using RBC lysis buffer (Sigma) as per the manufacturer’s protocol. The isolated splenocytes were cultured with RPMI 1640 medium containing 10% FBS and 1% penicillin-streptomycin in 6-well plates. Subsequently, the cells in the individual wells were stimulated with 5 μg/mL of purified recombinant proteins for 48 h at 37°C with 5% CO_2_. Brefeldin A (5 μg/mL; Sigma) was added to the cells during the last 4 h of culture. Next, the splenocytes were washed with PBS by centrifugation at 300 × *g* for 10 min at 4°C and stained with PerCP-conjugated anti-mouse CD8a, PE-conjugated anti-mouse CD4, and APC-conjugated anti-mouse IFN-γ antibodies (BioLegend) for 1 h at room temperature in the dark. For CD4 and CD8a markers, surface staining was performed while IFN-γ was stained intracellularly. Cells were fixed using ice-cold acetone and methanol in a 1:1 ratio, followed by permeabilization using 0.01 percent of Triton-X 100 (Sigma) in PBS. Subsequently, the cells were subjected to flow cytometry analysis using LSRFortessa (BD Biosciences). The lymphocytes were gated based on the scattering profiles of stains, and the percentage population of CD4 and CD8 positive cells as well as IFN-γ producing cells, were quantified.

### Immunization of mice and challenge studies with *Brucella melitensis*

Mice were immunized with serodominant proteins, *viz*. BMEI0856, BMEI0063, BMEI1513, BMEI0748, or adjuvant alone, as described before. The mice injected with recombinant L7/L12 (BMEI0748) protein were considered the positive control group, while the mice injected with adjuvant alone were considered the negative control group. Forty-five days post-immunization, mice were challenged with *B. melitensis* 16 M. *B. melitensis* 16 M was grown in *Brucella* broth at 37° C until the OD reached 1 and resuspended at 2 × 10^6^ CFUs/ml in 100 μL of sterile PBS. Subsequently, mice were administrated intraperitoneally (i.p.) with 2 × 10^6^ CFU of *B. melitensis* 16 M. The mice were euthanized 2 weeks post-challenge, followed by aseptic removal of spleens for the colony forming unit (CFU) enumeration. The harvested spleens were homogenized individually in 1 mL sterile PBS using 1.0 mm zirconium beads (Benchmark Scientifics) for 60 s in a bead beater (Benchmark Scientifics). The homogenized spleens were 10-fold serially diluted and plated on *Brucella* agar plates in triplicates. The plates were incubated at 37°C with 5% CO_2_, and the number of CFU was counted after 3 days of incubation. The CFU results were represented as the mean log CFU ± SD per group.

### Development of Dps protein-based serodiagnostic assays

To develop Dps protein-based iELISA, the antigen concentration was optimized by performing checkerboard titrations. Various concentrations (10, 5, 2.5, 1.25, 0.625, 0.3125, 0.15625, 0.078125 μg/well) of Dps protein were coated in the 96-well microtiter plates and incubated overnight at 4° C. Subsequently, the coated plates were washed three times with 1 X PBST, followed by blocking with 300 μL of blocking buffer (1% BSA for human sera and 5% BSA for livestock sera) per well. The plates were incubated for 1 h at room temperature and given a wash as described earlier. Next, the brucellosis positive and negative sera (1:100 dilution) were added to the wells, and the plates were incubated for 2 h at room temperature. Next, the plates were washed four times with the wash buffer, and 100 μL of the HRP-conjugated anti-bovine IgG diluted at 1:5,000 (ThermoFisher) was added per well. The plates were incubated for 1 h at room temperature, followed by four washes and the addition of the TMB substrate solution (ThermoFisher). The plates were incubated for 10–15 min, followed by reading absorbance at 450 nm using an ELISA reader (PerkinElmer). For assessing the DIVA capability of Dps-based iELISA, the plates were coated with 100 ng of Dps protein per well, followed by iELISA as described above using serum samples collected at 21, 45, and 90 days post-*B. abortus* S19 vaccination. To evaluate the efficiency of Dps-based iELISA, human and cattle serum samples were screened using Dps-based iELISA, and the results were compared with that of commercially available, validated iELISA for human (NovaLisa) and cattle (PrioCheck) and RBPT. *Brucella*-culture positive/negative sera (bovine and mouse) and OIE-brucellosis positive/negative bovine reference serum samples were also screened using the Dps-based iELISA.

*Y. enterocolitica* O:9 immune and healthy rabbit serum samples were obtained from the Translational Research Platform for Veterinary Biologicals (TRPVB). Dps-based iELISA was performed with *Y. enterocolitica* O:9 immune and healthy rabbit serum samples as described before and HRP-conjugated anti-rabbit IgG at 1:5000 (CST) dilution was used as the secondary antibody. To perform iELISA with *Salmonella* immune serum, the sera were raised in mice against formalin-inactivated *S. typhimurium*. In brief, 6–8 weeks old female BALB/c mice were injected with 100 μL of inactivated *S*. typhimurium (1 × 10^5^) intraperitoneally. Serum samples were collected on day 21, followed by performing Dps-based iELISA as described before. The HRP-conjugated anti-mouse IgG at a dilution of 1:5,000 (Novus Biologicals) was used as the secondary antibody for iELISA.

To develop the prototype LFA, purified Dps protein and biotinylated bovine serum albumin (Abcam) were dispensed (1.5 mg/mL) as test and control lines, respectively on the nitrocellulose membrane (mdi Membrane Technologies) using a flow dispenser (mdi Membrane Technologies). The protein G-gold (Abcam) and streptavidin-gold (Abcam) conjugates were mixed in a 4:1 ratio and coated on the pre-treated conjugate pad. Various components of LFA were assembled with the help of commercial source. To evaluate the prototype LFA, the serum samples (10 μL) were applied to the sample applicator port, followed by 200 μL of chase buffer to facilitate the sample migration toward the conjugate pad. Antibodies in the sample bound to the protein G-gold continue traveling up the nitrocellulose membrane. Anti-Dps antibodies in the samples were then bound to the Dps protein on the test line, producing a visible test line signal by an accumulation of gold particles on the test line. Streptavidin-gold conjugate bound to BSA-biotin on the control line produced a signal irrespective of the presence of anti-Dps antibodies in the samples. The absorbent pad absorbed excess liquid. The individual test was considered positive when a clear test line was visible or negative when only a control line was observed. To evaluate the LFA, 10 pooled brucellosis positive/negative/S19-vaccinated field serum samples of cattle, goat and humans were tested.

The formula used for the evaluation is given below:

Sensitivity = a/(a + c) × 100.

Specificity = d/(b + d) × 100.

Accuracy = a + d/(a + b + c + d) × 100.

a - Number of serum samples positive both by DPS ELISA and RBPT/iELISA.

b - Number of serum samples negative by RBPT/iELISA, but positive by DPS ELISA.

c - Number of serum samples positive by RBPT/iELISA, but negative by DPS ELISA.

d - Number of serum samples negative both by DPS ELISA and RBPT/iELISA.

Cut-off for commercial LPS based iELISA kit (as per manufacturer’s instruction):

Per cent positivity (PP) = (OD_45o test sample_/mean OD_450 positive control_) × 100.

Per cent positivity (PP) >40% is brucellosis positive.

Per cent positivity (PP) <40% is brucellosis negative.

Cut-off for DPS-based iELISA kit:

Cut-off = (mean OD of confirmed negative samples) ± 3 Standard Deviation.

### Statistical analysis

The GraphPad Prism 6.0 software was used for the statistical analysis of experimental data. Data are shown as mean ± SD. Statistical significance was determined by one-way analysis of variance (ANOVA) for analyzing the data that involved more than two samples. FACS data were analyzed using the software FACS Diva (BD Biosciences). The positivity of the serum samples for Dps-based iELISA was determined by the mean OD greater than 2 standard deviations over the mean OD of the negative control. The specificity and sensitivity of Dps-based iELISA were calculated using Bayesian model statistics.

## Results

### High-throughput immunoprofiling of *Brucella melitensis* protein microarray identified serodominant proteins of *Brucella*

**Figure 1:**
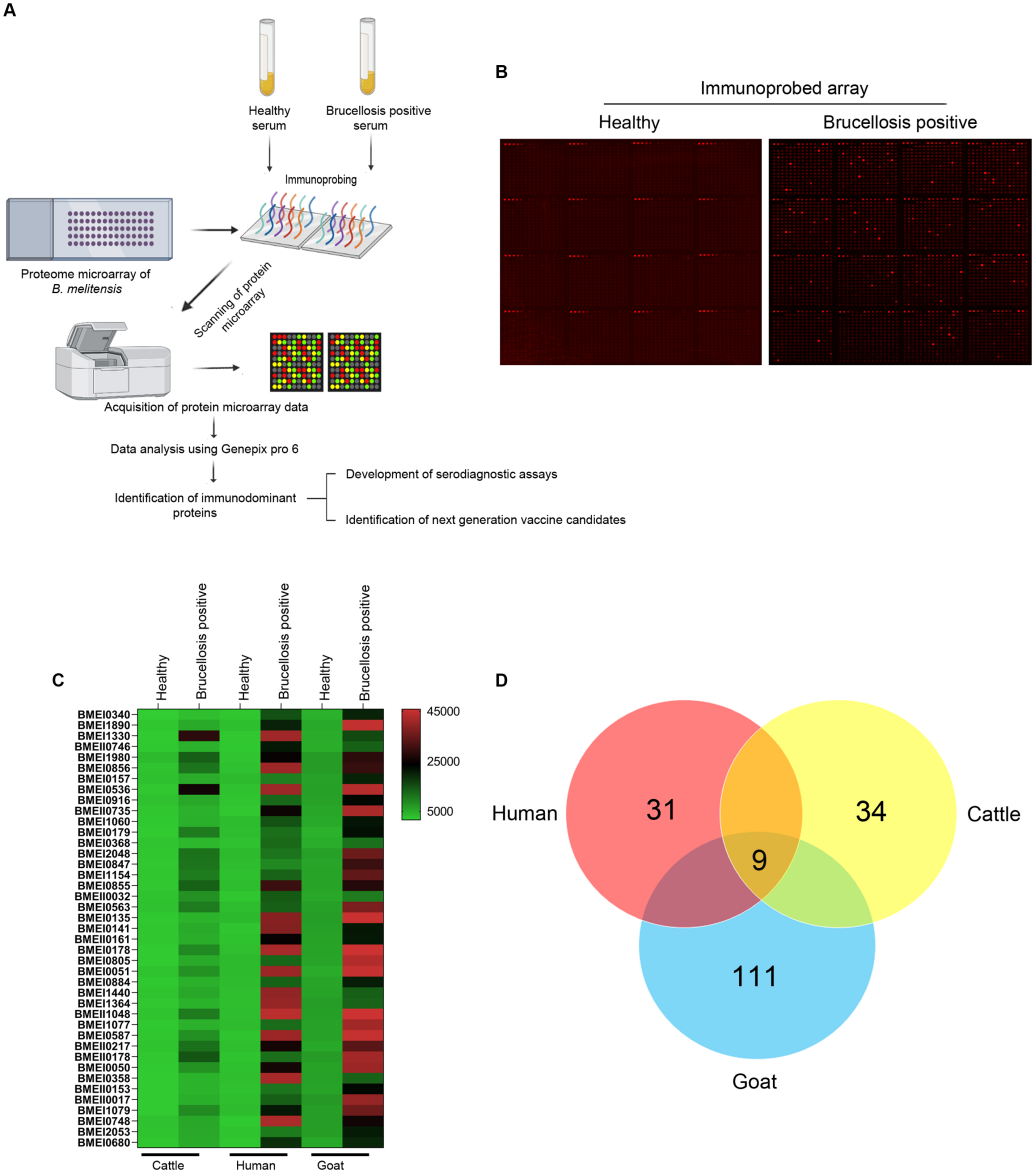
**(A)** A graphical abstract summarizing the identification process of serodominant protein antigens of *Brucella.* **(B)** Representative image of *B. melitensis* protein microarray immunoprobed with healthy or brucellosis-positive serum samples. The red spots on the array probed with healthy serum sample indicate the positive controls. The red spots on the array probed with brucellosis positive serum sample indicate the serodominant proteins that reacted with the respective antibodies in the serum. The intensity of red spots indicates the extent of seropositivity. Confirmed single strong positive or healthy serum samples from cattle, human and goat were utilized for immunoprobing that was performed in triplicates. **(C)** Heat map showing differential detection of serodominant proteins by healthy or brucellosis positive human, cattle and goat serum samples. The seroreactive *Brucella* proteins are mentioned in rows and the columns represent the host species. The seroreactivity of proteins is represented as varying intensity of red (strongest) to bright green (weakest). **(D)** The Venn diagram showing the number of *Brucella* proteins that reacted with serum samples from single or multiple host species. The number of seroreactive proteins detected in each species is shown in individual circle, while the number of proteins that are shared among the different species are shown in the intersection area.

High-throughput immunoprobing of microbial proteins facilitates the identification of serodominant antigens, which can be employed to develop vaccines and serodiagnostic assays. To identify and characterize the serodominant protein antigens of *Brucella*, we performed a high-throughput immunoprobing of the *B. melitensis* protein microarray using serum samples from healthy or brucellosis-positive cattle, goat, and human (Figure 1A). The commercially available full proteome microarray that contains ~3,000 proteins of *B. melitensis* was used for immunoprofiling. The brucellosis-positive serum samples that harbor antibodies against the serodominant proteins of *Brucella* reacted with the respective proteins on the microarray (Figure 1A). The seroreactive proteins were detected by scanning the immunoprobed protein microarrays. The healthy human serum samples did not cross-react with any *Brucella* proteins on the array. However, we observed the reactivity of ELISA and RBPT-negative cattle and goat serum samples with some proteins on the array. Immunoprobing with brucellosis-positive serum samples of human, cattle, and goat detected various proteins on the array (Figure 1B). Subsequently, the scanned images of arrays were analyzed for data acquisition and identification of seroreactive proteins. The antigens with a signal intensity of more than two standard deviations over the mean of signal intensities from negative controls were defined as serodominant antigens (Liang et al., 2010). The high-ranking serodominant proteins from individual species are illustrated as a heat map (Figure 1C). Among the 3,000 antigenic proteins screened, a set of 40, 43, and 120 antigens reacting with human, cattle and goat serum samples, respectively, were identified serodominant (Figure 1D; Tables 2–4). The analysis also identified a set of 9 antigens that were equally seroreactive with all the species (Figure 1D; Table 5).

**Table 2:** The list of identified serodominat proteins that reacted with human sera.

Peptidoglycan-associated lipoprotein	BMEI0340
Transporter	BMEI1890
Protease Do	BMEI1330
Branched-chain alpha-keto acid dehydrogenase subunit E2	BMEII0746
Isopropylmalate isomerase large subunit	BMEI0157
Immunogenic protein	BMEI0536
Hypothetical protein	BMEI0916
Periplasmic oligopeptide-binding protein precursor	BMEII0735
Hypothetical protein	BMEI1060
Hypothetical protein	BMEI0179
Hypothetical protein	BMEI0368
ATP-dependent protease ATP-binding subunit HslU	BMEI2048
Preprotein translocase subunit SecG	BMEI0847
NADH dehydrogenase subunit E	BMEI1154
Pyruvate dehydrogenase subunit beta	BMEI0855
VirB8	BMEII0032
Hypothetical protein	BMEI0563
Outer membrane lipoprotein	BMEI0135
Dihydrolipoamide succinyltransferase	BMEI0141
Flagellar hook-associated protein FlgL	BMEII0161
Hypothetical protein	BMEI0178
Hypothetical protein	BMEI0805
Hypothetical protein	BMEI0051
DNA gyrase subunit A	BMEI0884
Thiol:disulfide interchange protein DsbA	BMEI1440
Transcriptional regulatory protein MUCR	BMEI1364
Molecular chaperone GroEL	BMEII1048
Hypothetical protein	BMEI1077
COML competence lipoprotein	BMEI0587
Periplasmic dipeptide transport protein precursor	BMEII0217
DNA starvation/stationary phase protection protein Dps	BMEI1980
High-affinity zinc uptake system protein ZNUA	BMEII0178
CobT protein	BMEI0050
Deoxyuridine 5′-triphosphate nucleotidohydrolase	BMEI0358
Hypothetical protein	BMEII0153
Outer membrane lipoprotein	BMEII0017
Lipoprotein NlpD	BMEI1079
50S ribosomal protein L7/L12	BMEI0748
Transporter	BMEI2053
Preprotein translocase subunit SecD/SecF	BMEI0680

**Table 3:** The list of identified serodominant proteins that reacted with goat sera.

Transporter	BMEI1890
30S ribosomal protein S16	BMEI0227
Hypothetical protein	BMEI1866
Immunogenic protein	BMEI0536
Hypothetical protein	BMEI0810
Protease Do	BMEI0613
Type I restriction-modification enzyme S subunit	BMEII0452
Preprotein translocase subunit SecA	BMEI0121
HlyD family secretion protein	BR_1060
Periplasmic oligopeptide-binding protein precursor	BMEII0735
Nucleoside triphosphate pyrophosphohydrolase	BMEI0920
Hypothetical protein	BMEI1847
Hypothetical protein	BMEII0073
Flagellin	BMEII0150
Hypothetical protein	BMEI0699
Cell surface protein	BMEI1872
Riboflavin synthase subunit beta	BMEII0589
Sensory transduction histidine kinase	BMEI0370
2-oxoisovalerate dehydrogenase subunit alpha	BMEII0748
ATP-dependent protease ATP-binding subunit HslU	BMEI2048
ATPase	BMEI1370
Cystine-binding periplasmic protein precursor	BMEII0601
Multidrug resistance protein A	BMEII1118
Leu/Ile/Val-binding protein precursor	BMEII0103
Preprotein translocase subunit SecG	BMEI0847
acyl-CoA hydrolase	BMEI0503
LemA protein	BMEI0228
Electron transfer flavoprotein-ubiquinone oxidoreductase	BMEI1320
NADH dehydrogenase subunit E	BMEI1154
Hypothetical protein	BMEII0924
Sugar-binding protein	BMEII0755
Catalase	BMEII0893
Hypothetical protein	BMEI0563
Hypothetical protein	BMEII0231
Thioredoxin	BMEII0401
trbJ protein	BR_A0368
Outer membrane lipoprotein	BMEI0135
Monovalent cation/H+ antiporter subunit G	BMEII0765
Hypothetical protein	BMEI0973
Colicin V production protein	BMEI1487
Molecular chaperone DnaJ	BMEI1513
Cysteine synthase A	BMEI0101
Alpha-methylacyl-CoA racemase	BMEII1076
Non-motile and phage-resistance protein	BMEI0417
ABC transporter substrate-binding protein	BMEII0338
Superoxide dismutase (Cu-Zn)	BMEII0581
Copper-containing nitrite reductase precursor	BMEII0988
Hemagglutinin	BMEII0717
Hypothetical protein	BMEI1000
Hypothetical protein	BMEI0178
Hypothetical protein	BMEI0805
Osmotically inducible protein C	BMEII0409
Acetyl-CoA carboxylase biotin carboxyl carrier protein subunit	BMEI1062
Translaldolase	BMEI0244
Dihydroxyacetone kinase	BMEI0397
Dihydrolipoamide dehydrogenase	BMEI0145
Flagellar motor protein MotB	BMEII0154
Oligopeptide-binding protein appa precursor	BMEII0859
Membrane-bound lytic murein transglycosylase B	BMEI0223
Hypothetical protein	BMEI0051
Hypothetical protein	BMEI0063
Alkaline phosphatase	BMEI0790
Hypothetical protein	BMEI1865
tRNA (guanine-N(1)-)-methyltransferase	BMEI0149
Ribosome biogenesis GTP-binding protein YsxC	BMEII0274
Membrane metalloprotease	BMEI0829
Cytoplasmic protein	BMEII0772
D-ribose-binding periplasmic protein precursor	BMEI1390
Molecular chaperone GroEL	BMEII1048
Flagellar motor switch protein FLIM	BMEII1110
ABC transporter periplasmic-binding protein	BMEII0702
Sugar-binding protein	BMEII0590
Hypothetical protein	BMEI1514
Invasion protein B	BMEI1584
Hypothetical protein	BMEI0060
MarR family transcriptional regulator	BMEII0311
Flagellar basal-body rod protein FlgB	BMEII1089
Hypothetical protein	BMEI1077
Oxidoreductase	BMEI1710
Acetylornithine transaminase protein	BMEI1621
Inner membrane protein translocase component YidC	BMEII0275
Flagellar basal body rod modification protein	BMEII0164
D-ribose-binding periplasmic protein precursor	BMEII0435
Hypothetical protein	BMEII0989
COML competence lipoprotein	BMEI0587
Periplasmic protein of efflux system	BMEI0653
ABC transporter substrate-binding protein	BMEI0015
DNA mismatch repair protein MutS	BMEI1801
Translocation protein TolB	BMEI0339
Hypothetical protein	BMEI0186
Hypothetical protein	BMEII0468
3-oxoacyl-ACP synthase	BMEI1112
Stomatin like protein	BMEII0019
Periplasmic dipeptide transport protein precursor	BMEII0217
DNA starvation/stationary phase protection protein Dps	BMEI1980
Ferric uptake regulation protein	BMEI0375
Glycolate oxidase subunit GLCD	BMEI1527
High-affinity zinc uptake system protein ZNUA	BMEII0178
Hypothetical protein	BMEI1724
Leucine- isoleucine- valine- threonine- and alanine-binding protein precursor	BMEI1930
CobT protein	BMEI0050
AsnC family transcriptional regulator	BMEI0357
Outer membrane lipoprotein	BMEII0017
Hypothetical protein	BMEI0641
ABC transporter substrate-binding protein	BMEI1954
Carnitine operon oxidoreductase CaiA	BMEI0848
Multidrug resistance protein A	BMEI0926
50S ribosomal protein L7/L12	BMEI0748
Hypothetical protein	BMEI0651
Lipoprotein NlpD	BMEI1079
Cell division protein FtsZ	BMEI0585
Dithiobiotin synthetase	BMEII0777
D-3-phosphoglycerate dehydrogenase	BMEII0813
Hypothetical protein	BMEII0895
Glycerol-3-phosphate-binding periplasmic protein precursor	BMEII0625
Hypothetical protein	BMEI0796
Phosphate-binding periplasmic protein	BMEI1989
Hypothetical protein	BMEI1695
Short-chain dehydrogenase/reductase	BMEI1832
Cysteine desulfurase	BMEI1042

**Table 4:** The list of identified serodominant proteins that reacted with cattle sera.

Protease Do	BMEI1330
bifunctional N-acetylglucosamine-1-phosphate uridyltransferase/glucosamine-1-phosphate acetyltransferase	BMEII0684
Immunogenic protein	BMEI0536
Hypothetical protein	BMEI0810
Protease Do	BMEI0613
tRNA pseudouridine synthase B	BMEI1963
Membrane fusion protein MTRC	BMEI0892
Hypothetical protein	BMEI0179
Cell surface protein	BMEI1872
Riboflavin synthase subunit beta	BMEII0589
2-oxoisovalerate dehydrogenase subunit alpha	BMEII0748
ATP-dependent protease ATP-binding subunit HslU	BMEI2048
Succinyl-CoA synthetase subunit beta	BMEI0138
Aliphatic sulfonates-binding lipoprotein	BMEII0109
Iron(III)-binding periplasmic protein precursor	BMEII1120
Multidrug resistance protein A	BMEII1118
Preprotein translocase subunit SecG	BMEI0847
Hypothetical protein	BMEII0010
Acyl-CoA hydrolase	BMEI0503
Leucine- isoleucine- valine- threonine- and alanine-binding protein precursor	BMEI0263
Cytochrome C-type biogenesis protein CYCH	BMEI1334
Hypothetical protein	BMEII0913
50S ribosomal protein L4	BMEI0758
NADH dehydrogenase subunit E	BMEI1154
Ribosomal-protein-serine acetyltransferase	BMEII0002
Pyruvate dehydrogenase subunit beta	BMEI0855
Hypothetical protein	BMEII0924
Hypothetical protein	BMEI0563
Hypothetical protein	BMEI1033
Molecular chaperone DnaJ	BMEI1513
Hemagglutinin	BMEII0717
Hypothetical protein	BMEI0119
Nitrogen fixation protein FIXG	BMEI1567
Hypothetical protein	BMEI0063
Molecular chaperone GroEL	BMEII1048
ABC transporter periplasmic-binding protein	BMEII0702
Peptidyl-prolyl cis-trans isomerase	BMEI0123
Hypothetical protein	BMEI0443
Periplasmic dipeptide transport protein precursor	BMEII0217
DNA starvation/stationary phase protection protein Dps	BMEI1980
High-affinity zinc uptake system protein ZNUA	BMEII0178
Phosphate-binding periplasmic protein	BMEI1989
Molybdopterin (MPT) converting factor subunit 1	BMEI1253

**Table 5:** The list of identified serodominant antigens that reacted with sera from both animals and humans.

Protease Do	BMEI1330
Immunogenic protein	BMEI0536
ATP-dependent protease ATP-binding subunit HslU	BMEI2048
NADH dehydrogenase subunit E	BMEI1154
Molecular chaperone GroEL	BMEII1048
Periplasmic dipeptide transport protein precursor	BMEII0217
DNA starvation/stationary phase protection protein Dps	BMEI1980
High-affinity zinc uptake system protein ZNUA	BMEII0178
Pyruvate dehydrogenase complex dihydrolipoamide acetyltransferase	BMEI0856

### Cloning, expression, and purification of selected serodominant proteins of *Brucella*

**Figure 2:**
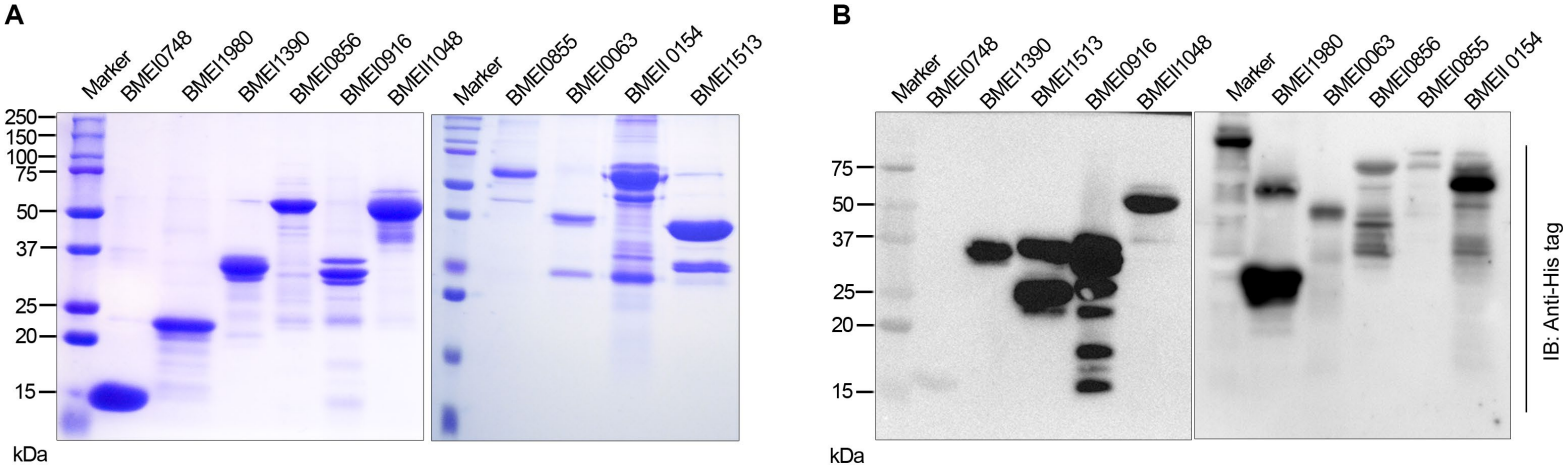
**(A)** SDS-PAGE gel image of purified serodominant proteins. The selected serodominant proteins were cloned and overexpressed in *E. coli* with a C-terminal 6X His-tag. Subsequently, the overexpressed proteins were purified using Ni-NTA affinity chromatography. **(B)** Immunoblot showing the purified serodominant proteins. The blot was probed with HRP-conjugated anti-His antibody to detect the His-tagged proteins.

We identified many serodominant proteins of *Brucella* using high-throughput immunoprobing of the *B. melitensis* protein microarray. To characterize these antigens further, we selected 10 serodominant proteins based on their seroreactivity and novelty. These proteins are either shared by brucellosis-positive animals and humans or uniquely expressed by individual species (Table 6). The vaccine and diagnostic potentials of some of these serodominant proteins were not explored before. The coding sequences of respective proteins were amplified from the chromosomal DNA of *B. melitensis*, followed by cloning them into a pET21a(+) prokaryotic expression vector that harbors a C-terminal 6X His tag. Subsequently, the clones were confirmed by restriction enzyme digestion and sequencing. To overexpress serodominant proteins, pET21a(+) harboring the *Brucella* genes were introduced into BL21DE3 *E. coli.* The overexpression of recombinant *Brucella* proteins was induced in *E. coli* using IPTG, followed by analyzing the expression levels by SDS-PAGE (Supplementary Figure S1). Next, the overexpressed *Brucella* proteins were purified using Nickel-NTA-agarose affinity chromatography, and the purity of the proteins was assessed by SDS-PAGE (Figure 2A). Further, we examined the purified *Brucella* proteins by immunoblotting using an anti-His-tag antibody (Figure 2B). The immunoblot showed overexpressed *Brucella* protein of expected size that further confirmed the identity of the recombinant proteins.

**Table 6:** The list of 10 high-ranking serodominant proteins that were selected for the study.

ID	Gene name
BMEI1980	DNA starvation/ stationary phase protection protein Dps (common in all the species)
BMEI1390	D-ribose-binding periplasmic protein precursor (detected in goat sera)
BMEI1513	J domain-containing protein (detected in goat sera)
BMEI0063	Hypothetical protein (detected only in animal sera)
BMEI0856	Pyruvate dehydrogenase complex dihydrolipoamide acetyltransferase (common in all the species)
BMEI0916	Hypothetical protein (detected in human sera)
BMEII1048	Molecular chaperone GroEL (common in all the species)
BMEI0855	Pyruvate dehydrogenase subunit beta (detected in human and cattle sera)
BMEII0154	MotB family protein (detected in goat sera)
BMEI0748	50S ribosomal protein L7/L12 (detected in human sera and goat sera)

### Evaluation of the immunogenicity of purified sero-dominant proteins of *Brucella* in mice

**Figure 3:**
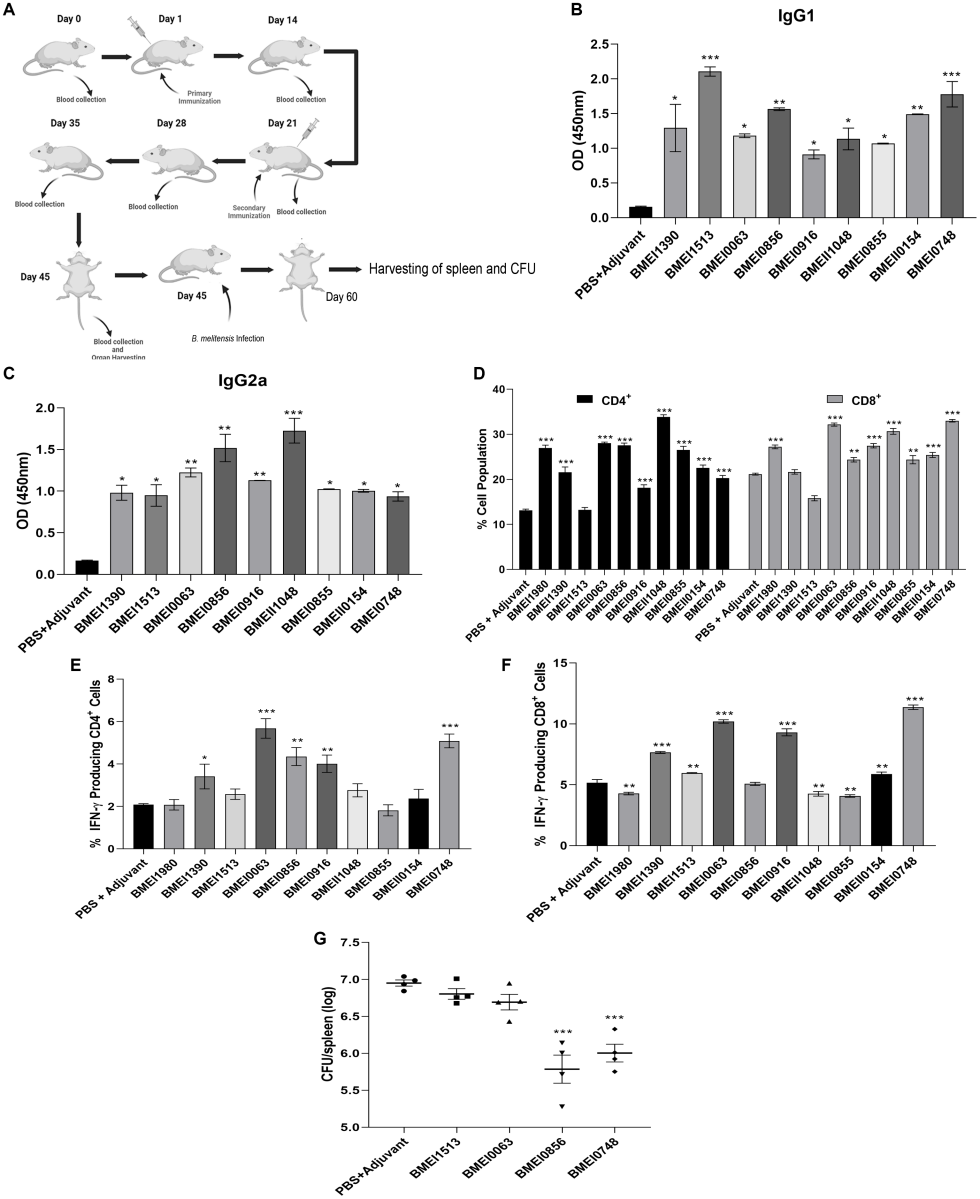
**(A)** A pictographic representation of the study to examine the immunogenicity of purified serodominant proteins of *Brucella.* BALB/c mice were immunized with purified. Recombinant proteins on day 1, followed by administering a booster dose on day 21. Blood samples were collected on day 0, 14, 21, 28, 35, and 45 post-immunization. Mice were euthanized on day 45, followed by collection of spleen. For the challenge studies, the immunized mice were infected with *B. melitensis* on day 45 and euthanized on day 60, followed by collection of spleen for the CFU enumeration. **(B,C)** ELISA showing the levels of IgG1 and IgG2a, respectively in the serum samples. The serum samples collected from immunized mice on day 45 were subjected to ELISA to determine the levels of IgG1 and IgG2a. Mice injected with PBS+Adjuvant and BMEI0748 (L7/L12) were used as the negative and positive controls, respectively. **(D)** Percent cell population of CD4^+^ and CD8^+^ T cells, in the immunized or control mice. The splenocytes were isolated from the immunized mice, followed by treating the cells with respective serodominant proteins. Forty-eight hours post-treatment, the cells were stained with anti-CD4, and anti-CD8 antibodies conjugated with fluorescent dyes, followed by FACS analysis. **(E,F)** Showing percent cell population of IFN-γ producing CD4^+^ and CD8^+^ T cells in or control mice. The staining procedure was followed as mentioned above followed by staining with anti-CD4, -CD8 and -IFN-γ antibodies conjugated with fluorescent dyes, followed by FACS analysis. **(G)** Bacterial load in the spleen of mice immunized with the serodominant proteins, BMEI0856, BMEI0063, BMEI1513, BMEI0748 (L7/L12) or adjuvant alone. The immunized mice were challenged with 2 × 10^6^ CFUs/ml of *B. melitensis* 16 M, followed by enumeration of CFU at 14 days post-infection. Each data point represents individual mice per group and the bar indicates average of the group. Mice injected with PBS + Adjuvant and BMEI0748 were used as the negative and positive controls, respectively. All the data are presented as mean ± SD. (*p < 0.05; **p < 0.01; ***p < 0.001).

The reactivity of *Brucella* proteins with brucellosis-positive serum samples suggests that these proteins are secreted or released by *Brucella* into the circulatory system of the host, resulting in the formation of antibodies against them. Since these proteins are immunogenic, they may induce *Brucella*-specific immune responses other than antibody generation in the host. To examine this, we analyzed the immunogenicity of purified *Brucella* proteins in mice. The mice were immunized with serodominant proteins, followed by the collection of blood samples at various days post-immunization (Figure 3A). We used the immunogenic protein of *Brucella*, L7/L12 (BMEI0748) as the positive control as this antigen has been reported to induce a protective immune response in brucellosis (Oliveira et al., 1994; Oliveira and Splitter, 1994, 1996; Singh et al., 2015). The mice were sacrificed at the end of the experiment, followed by analyzing various cell populations to determine the induction of cell-mediated immune responses. Serum samples collected on different days were analyzed for IgG1 and IgG2a levels to estimate the humoral immune response induced by these antigens (Supplementary Figure S2). We observed a high antibody titer in the immunized mice, indicating that these antigens are potent inducers of the humoral immune response. IgG1 and IgG2a are the markers of Th2 and Th1 responses, respectively, in mice where the Th1 response is reported to be important for protection against brucellosis (Golding et al., 2001; Yingst and Hoover, 2003; Skendros et al., 2011; Vitry et al., 2014). We observed a high titer of IgG2a antibody with BMEI1980, BMEI0063, BMEI0856, BMEI0916, BMEII1048, and BMEI0855 indicating a Th1 specific response (Figure 3C). In contrast, the *Brucella* proteins, BMEI1513, BMEI0748, BMEI1390, and BMEII0154 exhibited induction of IgG1 response, suggesting a Th2 immune response (Figure 3B).

Next, we analyzed other indicators of protective immunity against *Brucella,* such as the cell population producing IFN-γ and the levels of CD4^+^ and CD8^+^ T cells. The splenocytes isolated from the immunized mice were treated with the respective serodominant proteins for 48 h, followed by staining the cells with PerCP-conjugated anti-mouse CD8a, PE-conjugated anti-mouse CD4, and APC-conjugated anti-mouse IFN-γ antibodies for flow cytometry analysis. A higher percentage of CD4^+^ and CD8^+^ T cells population was observed in the mice immunized with BMEI1980, BMEI0063, BMEI0856, BMEI0916, BMEII1048, BMEI0855, BMEII0154, and BMEI0748 compared to the adjuvant alone (Figure 3D). We also observed a high percentage of IFN-γ producing CD4^+^ and CD8^+^ T cells in mice immunized with BMEI1390, BMEI0063, BMEI0856, BMEI0916, BMEI0748 and BMEI1980, BMEI1390, BMEI1513, BMEI0063, BMEI0916, BMEII1048, BMEI0855, BMEII0154, BMEI0748, respectively (Figures 3E,F, Supplementary Figure S3).

### Evaluation of protective efficacy of serodominant proteins against *Brucella melitensis* challenge

Next, we performed a challenge study with *B. melitensis* using the mice immunized with four serodominant proteins *viz*. BMEI0856, BMEI0063, BMEI1513, and BMEI0748. Our immunogenicity studies revealed that the serodominant proteins, BMEI0856 and BMEI0063, induced a significant level of Th1 type immune response, whereas BMEI1513 induced a Th2 type response. The mice immunized with L7/L12 protein (BMEI0748) and adjuvant alone served as the positive and negative control groups, respectively. The immunized mice were challenged with *B. melitensis*, followed by harvesting the spleen and enumerating bacterial load at 2 weeks post-infection. The protection efficiency of a vaccine candidate is determined by the significant reduction in the number of bacterial colonies in the spleen of immunized mice compared to the control. The mice immunized with BMEI0856 showed a significant reduction of CFU, indicating the protective effect of this protein against *the B. melitensis* challenge (Figure 3G). As reported previously, L7/L12 also exhibited protective efficacy against challenge with *B. melitensis*. Interestingly, BMEI0856 immunized mice showed lesser bacterial load compared to L7/L12. On the other hand, no significant difference in the bacterial load was observed in the mice immunized with the BMEI1513 or BMEI0063 compared to the control groups (Figure 3G).

### The serodominant protein, Dps, reacts with brucellosis-positive serum samples and exhibits DIVA capability

**Figure 4:**
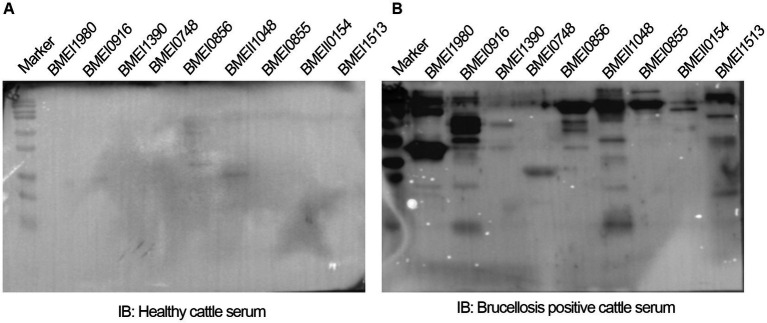
**(A,B)** Immunoblots showing the reactivity of purified serodominant proteins with healthy or brucellosis-positive cattle serum samples, respectively. The purified serodominant proteins were resolved on SDS-PAGE gel, followed by immunoblotting. The membranes were probed with healthy or brucellosis-positive serum samples, followed by HRP-conjugated anti-bovine IgG.

The positive signals from the immunoprobed protein array indicated the seroreactivity of serodominant proteins with the respective antibodies in the brucellosis-positive serum samples. To confirm this data further, we examined the seroreactivity of purified serodominant proteins by immunoblotting. The proteins were resolved on SDS-PAGE, followed by transferring them onto the PVDF membrane. Subsequently, the immunoblots were incubated with healthy or brucellosis-positive cattle serum samples. We observed seroreactivity of purified serodominant proteins with varying efficiency where BMEI1980 BMEI0916, BMEI0856, BMEII1048, BMEI0855, and BMEI1513 exhibited strong seroreactivity (Figure 4B). The healthy serum sample did not react with any of the purified proteins that we tested (Figure 4A).

**Figure 5:**
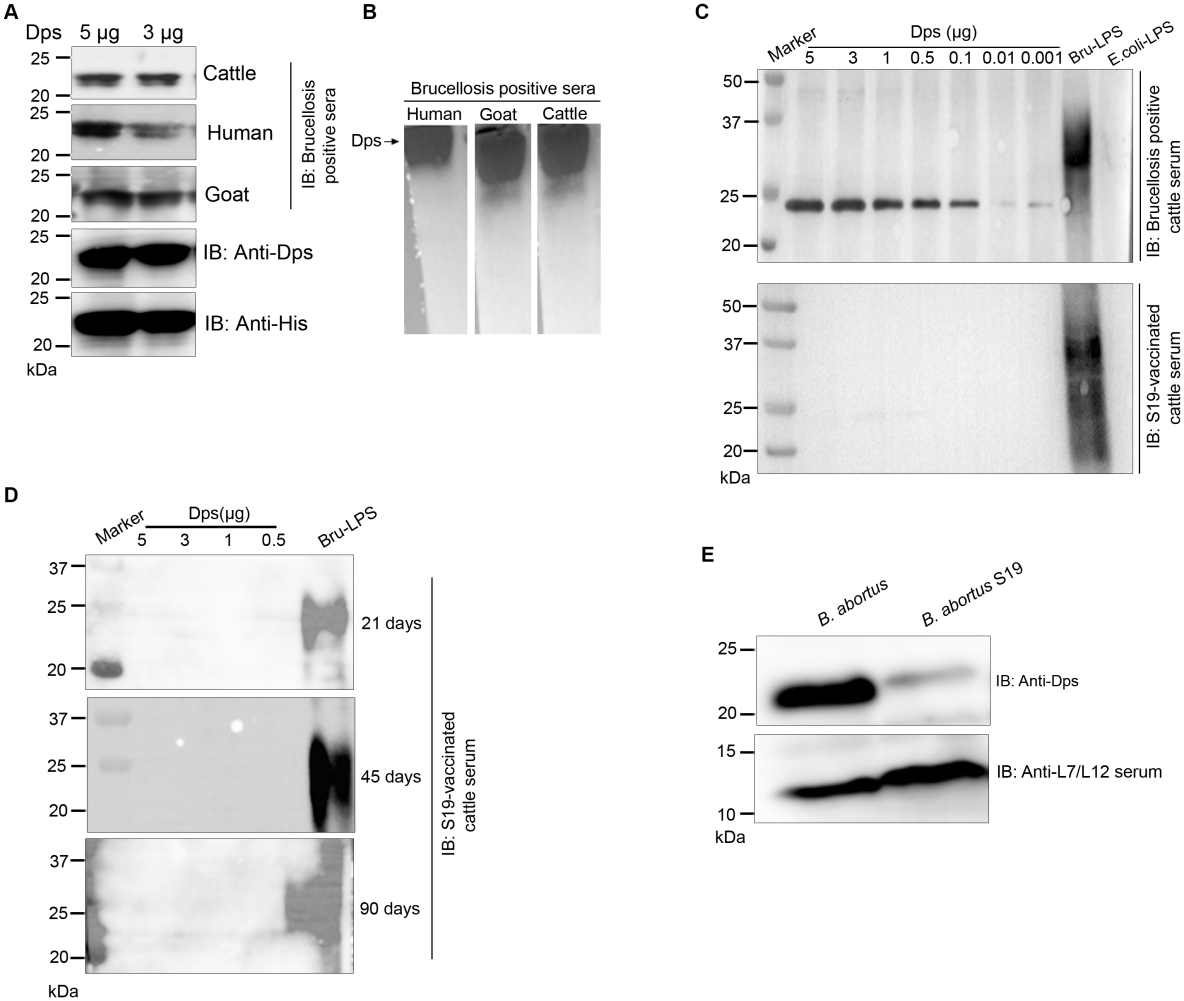
Seroreactivity of Dps protein with sera from human and animals. **(A,B)** Purified Dps protein resolved on SDS-PAGE and native-PAGE gel, respectively, followed by immunoblotting. The membrane was probed with 10 pooled healthy or brucellosis-positive serum samples from human, goat and cattle, followed by species-specific HRP-conjugated anti-IgG. The Dps protein was also confirmed by probing the blot with anti-His HRP antibody and anti-Dps antibody. **(C)** Immunoblot showing the reactivity of various concentrations (5, 3, 1, 0.5, 0.1, 0.01, and 0.001 μg) of Dps protein with 10 pooled serum samples from brucellosis-positive cattle or cattle vaccinated with *Brucella abortus* S19 strain. LPS of *B. abortus* S19 and *E. coli* were used as the positive and negative controls, respectively. **(D)** Immunoblot showing various concentrations (5, 3, 1, 0.5 μg) of Dps protein probed with 10 pooled cattle serum samples collected at 21, 45, and 90 days post-vaccination with *Brucella abortus* S19. The LPS of *B. abortus* S19 was used as the positive control. **(E)** Immunoblot showing the levels of Dps protein in *B. abortus* and *B. abortus* S19 strain. The total cell lysates of *B. abortus* or *B. abortus* S19 was subjected to immunoblotting, followed by probing the blot with anti-Dps antibody. The L7/L12 protein was detected on the blot using immunized serum from mice.

We observed strong reactivity of BMEI1980 with the brucellosis-positive serum samples of cattle and humans. The BMEI1980 encodes the DNA-binding protein from starved cells, Dps. A high throughput proteome analysis reported that the Dps protein is expressed only in the *B. abortus* 2,308 compared to the vaccine strain, *B. abortus* S19 (Lamontagne et al., 2009). However, there was no experimental validation of this observation. Therefore, we performed a detailed characterization of Dps protein, intending to develop improved serodiagnostic assays with DIVA capability. We analyzed the immuno-reactivity of purified Dps protein with serum samples from animals and humans. The immunoblots of Dps protein were probed with healthy sera or brucellosis-positive human, goat, cattle, or *B. abortus* S19-vaccinated cattle serum samples. The Dps protein was efficiently detected by brucellosis-positive serum samples from both animals and humans (Figures 5A,B). Further, we found that Dps protein failed to react with the serum sample from *B. abortus* S19-vaccinated cattle. To confirm the differential recognition of Dps protein, we performed immunoprobing of various concentrations of Dps protein with the sera from naturally infected or *B. abortus* S19-vaccinated cattle (Figure 5C). In agreement with previous data, the serum samples from *B. abortus* S19-vaccinated cattle did not detect Dps protein. We used LPS of *E. coli* and *B. abortus* as the negative and positive controls, respectively, for the immunoblots. Next, we probed the Dps blot with cattle serum samples collected at 21, 45, and 90 days post-*B. abortus* S19-vaccination. We did not observe any seroreactivity of Dps protein with serum samples after various days post-vaccination (Figure 5D). The LPS of *B. abortus* reacted with the sera, which confirms that the serum samples are derived from *B. abortus* S19-vaccinated animals.

Next, we examined the reason behind the non-reactivity of Dps protein with the *B. abortus* S19-vaccinated cattle sera. It is possible that the Dps protein was not expressed in the *B. abortus* S19 vaccine strain, resulting in a lack of antibody production against this protein in the vaccinated animals. To examine this, we performed immunoblotting with total lysates of *B. abortus* and *B. abortus* S19 strain. The membrane was probed with an anti-Dps antibody, which can detect the endogenous level of Dps protein. We used the reported serodominant protein, L7/L12, as the positive control. We observed a diminished level of Dps protein in the *B. abortus* S19 strain in comparison to the wild-type *B. abortus* (Figure 5E). In contrast, we observed a uniform expression of L7/L12 protein in *B. abortus* and *B. abortus* S19 vaccine strains (Figure 5E).

### Dps protein-based serodiagnostic assays exhibit DIVA capability and detect brucellosis in animals and humans

**Figure 6:**
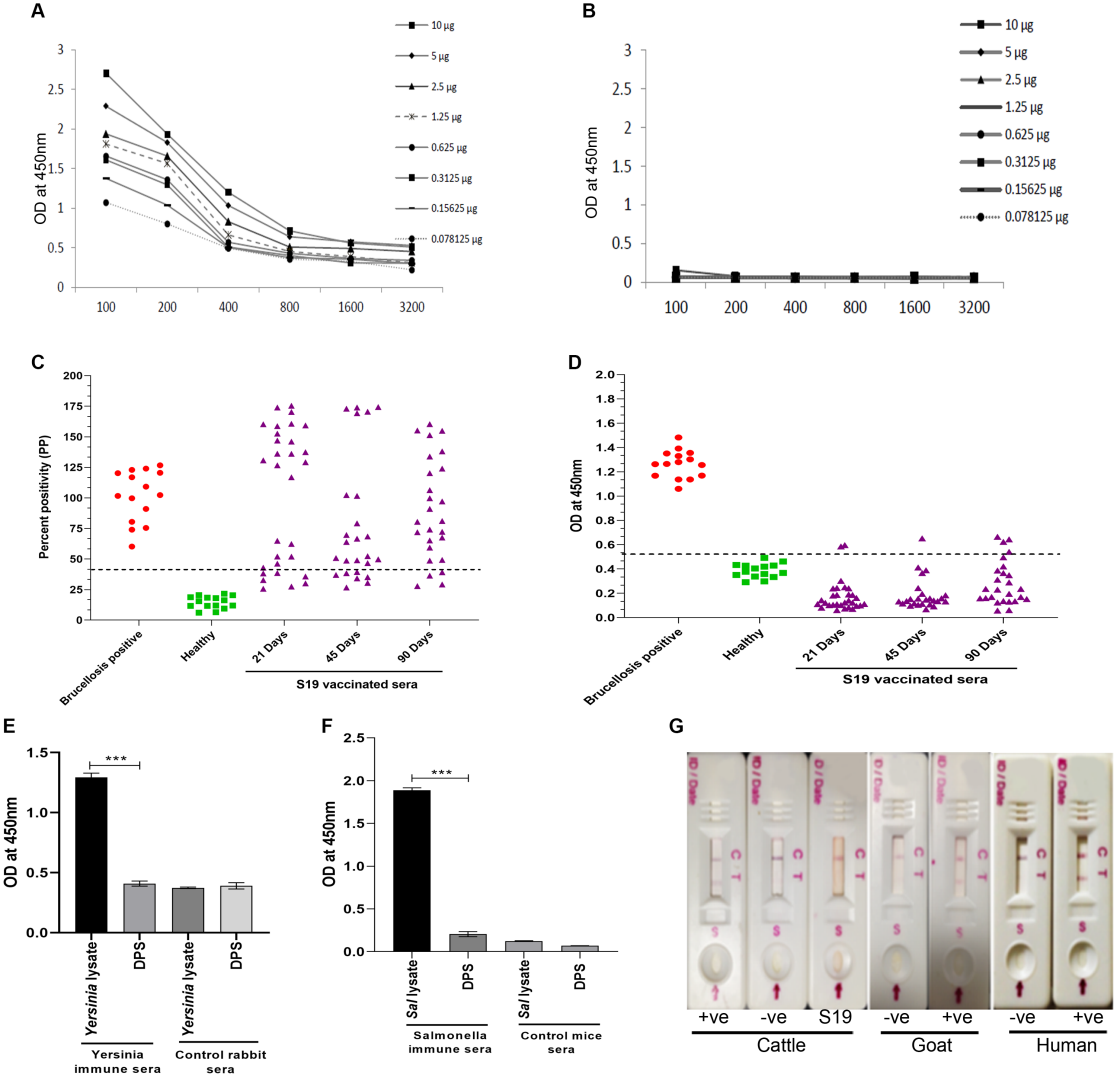
Development of Dps-based serodiagnostic assays. **(A,B)** Checkerboard titration analysis using brucellosis positive and healthy human serum samples, respectively, to determine the optimal concentration of Dps protein for ELISA development. **(C)** Percent positivity (PP) of healthy or brucellosis positive or *B. abortus* S19-vaccinated cattle serum samples analyzed using LPS-based iELISA kit (ProCheck). The S19-vaccinated serum samples collected at 21, 45, and 90 days post-vaccination were used for iELISA. Each data point indicates individual serum sample and the horizontal line shows the cut-off for iELISA. Samples with PP value 40% or above were considered positive as per the manufacturer’s instructions. **(D)** Dot plot showing OD values of Dps-based iELISA that was performed with brucellosis positive or negative or *B. abortus* S19-vaccinted serum samples. The S19-vaccinated serum samples collected on 21, 45 and 90 days post-vaccination were used for iELISA. The y-axis indicate OD at 450 nm and x-axis indicate number of serum samples screened. The horizontal line indicates the cut-off. Samples with OD values of 0.5 or below were considered negative. **(E)** Evaluation of cross-reactivity of Dps protein with *Yersinia* immune rabbit serum. *Yersinia* immune serum (*n* = 2) or control rabbit serum (*n* = 2) was screened by Dps-based iELISA or iELISA using *Yersinia* whole cell lysate as the antigen. The Y-axis shows the OD values at 450 nm and X-axis indicate the antigens screened with *Yersinia* immune or healthy rabbit serum samples. **(F)** Evaluation of cross-reactivity of Dps protein with *S. typhimurium* immune sera generated in mice. The iELISA was performed with Dps protein or whole cell lysate of *S. typhimurium* as the antigen. The OD values at 450 nm are shown on X-axis and Y-axis indicates the antigens screened using *S. typhimurium* immune serum (*n* = 2) or control mice serum samples (*n* = 2). **(G)** Detection of brucellosis in the animals and humans using Lateral Flow Assay (LFA) based on the Dps protein. The serum samples from healthy or brucellosis positive cattle, goat and human were used for testing the LFA. The absence of test line with serum samples from *B. abortus* S19-vaccinated cattle show the DIVA capability of the assay. C, Control line: T, test line.

Since both the animal and human sera efficiently recognized Dps protein, we wished to develop a lateral flow assay (LFA) and an indirect ELISA (iELISA) based on this protein. Toward developing iELISA, we determined the optimal concentration of Dps protein using the checkerboard titration assays (Figures 6A,B). Various concentrations of Dps protein were titrated against brucellosis-positive or negative human serum samples. The positivity of the serum samples was determined by the mean OD greater than two standard deviations over the mean OD of the negative controls (França et al., 2014). The cut-off value calculated for human serum samples was 0.45. The assay indicated that 1 μg/mL of Dps protein was optimal for developing the iELISA. To develop the assay further, various blocking agents and antibody dilutions were optimized. One percent BSA was found suitable for testing human serum samples, whereas 3% BSA was optimal for cattle serum samples. HRP-conjugated anti-bovine or anti-human antibody at a dilution of 1:5,000 was found optimal for Dps-based iELISA.

Next, the efficiency of Dps-based iELISA was compared with that of commercially available LPS-based iELISA or with RBPT. The validated LPS-based iELISA (NovaLisa) can detect brucellosis with human serum samples. Among the human serum samples tested, 26 sera were found to be positive by Dps protein-based iELISA, and 25 samples were positive by commercial iELISA. In contrast, only 14 samples were found positive by RBPT. A total of 195 samples were declared negative by all three tests. Both ELISAs had an agreement with 25 samples. Out of 207 samples, which were negative with RBPT, 11 samples were found positive by both ELISAs. In the case of one sample, there was a disagreement between the two ELISA (Table 7). The higher number of positive samples in iELISA than RBPT may suggest enhanced sensitivity of ELISA. Our in-house validation assays using human serum samples indicated 99% sensitivity and 98% specificity for the Dps-based iELISA (Table 8).

**Table 7:** Comparison of results obtained from Dps-based iELISA with RBPT or LPS-based iELISA using human serum samples.

Tests	Dps-based iELISA			
		Positive	Negative	Total
RBPT	Positive	14	0	14
	Negative	12	195	207
	Total	26	195	221
Indirect ELISA	Positive	25	0	25
	Negative	1	195	196
	Total	26	195	221

**Table 8:** Estimates obtained by Bayesian model statistical analysis of Dps-based and LPS-based iELISAs using human serum samples.

Estimates	Values
Sensitivity (%)	100 (86.28–100)
Specificity (%)	99.49 (97.19–99.99)
Accuracy	99.55 (97.50–99.99)
Positive predictive value	96.15 (77.97–99.44)
Negative predictive value	100
Positive likelihood ratio	196 (27.75–1384.52)
Negative likelihood ratio	0

We have also evaluated the seroreactivity of Dps-based iELISA using *Brucella*-culture positive/negative bovine and mouse sera and OIE-brucellosis positive/negative bovine reference serum samples (Supplementary Figures 4A,B). The assay indicated the detection of anti-Dps antibodies in the brucellosis positive reference serum samples. To examine the DIVA capability of iELISA, cattle serum samples were collected on various days post *B.* abortus S19 vaccination (21, 45, and 90 days) and were screened using the Dps-based or LPS-based iELISA. The OD values of the known positive, negative, and *B. abortus* S19-vaccinated serum samples were estimated, and the cut-off values were derived as described before. All the *B. abortus* S19-vaccinated serum samples were seropositive with LPS-based iELISA, whereas these sera showed OD values similar to negative controls with Dps-based iELISA (Figures 6C,D). The assay indicated that the Dps-based iELISA could differentiate *B. abortus* S19-vaccinated from naturally infected cattle.

The LPS-based serodiagnostic assays for brucellosis have been reported to cross-react with the serum samples of animals infected with *Y. enterocolitica* O:9, *S. typhimurium*, and *E. coli* (Al Dahouk et al., 2003a). Therefore, we examined the cross-reactivity of Dps-based iELISA with *Yersinia* and *Salmonella* immune sera. We did not observe any significant cross-reactivity with Dps-based iELISA against the *Yersinia* or *Salmonella* immune serum samples (Figures 6E,F). To further confirm the experimental data, we performed an immunoblot with whole cell lysates of *S. typhimurium, E. coli* or *B. abortus*, followed by probing the membrane with *Brucella* anti-Dps antibody. The anti-Dps antibody did not cross-react with any proteins in the lysates of *S. typhimurium* or *E. coli* other than detecting the endogenous Dps protein in the *B. abortus* lysate (Supplementary Figure S5). Collectively, our experimental data indicate that Dps-based iELISA can specifically detect brucellosis positive sera samples without showing any cross-reactivity.

Next, we developed a prototype LFA using Dps protein for pen-site application of this serodiagnostic assay. In the prototype LFA, Dps protein was used as the detection agent, while biotinylated bovine serum albumin was employed as the control. To examine the efficiency of LFA, healthy or brucellosis-positive humans, cattle, goat, and *B. abortus* S19-vaccinated cattle were added to the LFA device. A test line could be observed with brucellosis-positive animal and human serum samples (Figure 6G). In agreement with our previous observation, Dps protein did not react with the sera from *B. abortus* S19-vaccinated cattle. The healthy serum samples gave no test line indicating no cross-reactivity with the Dps protein (Figure 6G).

## Discussion

Brucellosis is an emerging zoonotic disease that seriously impacts human health and livestock productivity (Mcdermott et al., 2013; Rubach et al., 2013). The intracellular niche and the ability to subvert host immune responses enable *Brucella* to cause chronic infection in the host, making treatment options often difficult (Solera et al., 1997; Godfroid et al., 2011). Since there is no human vaccine for brucellosis, controlling the disease in animals through mass vaccination is the only viable option to limit human infections. Early detection and culling/quarantine of infected animals are crucial for controlling the spread of brucellosis in livestock. However, the major drawbacks of available animal vaccines and serodiagnostic assays pose serious hurdles in brucellosis control programs in various countries (Schurig et al., 2002; Galińska and Zagórski, 2013). Therefore, it is essential to identify and characterize novel serodominant antigens of *Brucella* to address major shortcomings of presently available vaccines and serodiagnostic assays.

Compared to other antigens, serodominant proteins are promising targets for developing improved diagnostic assays and vaccines. Once identified, these proteins can be expressed and purified from various expression systems making the process safer and cost-effective as large-scale culturing of the infectious pathogens and extraction of the antigens are not required. Serodiagnostic assays based on these recombinant proteins can provide higher sensitivity, specificity, and DIVA capability (Chaudhuri et al., 2010; Thavaselvam et al., 2010). These recombinant serodominant proteins could also serve as ideal candidates for developing safe and efficient vaccines for animal and human applications. Recent advancements in genomics and proteomics techniques permit large-scale screening for identifying serodominant proteins of pathogenic microorganisms (Liang et al., 2010). We used a full-proteome microarray of *B. melitensis* for the identification of serodominant proteins of *Brucella* from various host species. The immunoprofiling of *B. melitensis* proteins on the microarray yielded 202 potentially immunogenic proteins uniquely present or shared by multiple host species. Our analysis picked up many proteins of *Brucella*, such as BP26, ZunA, Cu-Zn SOD, Omp19, and Omp10, which were previously identified as serodominant and subsequently were used as candidate antigens for the development of either vaccines or serodiagnostic assays (Kim et al., 2004; Pasquevich et al., 2009; Sáez et al., 2012; França et al., 2014). These findings indicate the reliability of the experimental data generated in the present study. Subsequently, we evaluated the vaccine and diagnostic potential of 10 high-ranking immunogenic antigens, excluding previously reported well-characterized proteins.

We cloned, overexpressed, and purified the selected serodominant proteins, followed by analyzing their immunogenic potential to induce Th1 immune response against brucellosis in mice. Th1 type cellular immune response mainly involves generating IFN-γ producing T-cell populations, antigen-specific cytotoxic CD8+ T-cells, and a high titer of IgG2a is reported to confer protection in brucellosis (Golding et al., 2001; Yingst and Hoover, 2003; Skendros et al., 2011; Vitry et al., 2014). Mice immunized with BMEI1980, BMEI1390, BMEI0063, BMEI0856, BMEI0916, BMEII1048, BMEI0855, BMEII0154, and BMEI0748, showed higher percentage of CD4^+^, CD8^+^ cell population or IFN-γ producing CD4^+^, CD8^+^ cell population. Thus, suggesting a significant induction of Th1-type immune response. These proteins induced a high titer of IgG2a antibody, comparable to the response induced by the reported vaccine candidate, L7/L12 (Oliveira et al., 1994; Oliveira and Splitter, 1994, 1996; Singh et al., 2015). Switching antibody response to the IgG2a type could facilitate antibody-mediated phagocytosis of extracellular *Brucella*, which can enhance bacterial clearance (Ko and Splitter, 2003; Vitry et al., 2014). The identified serodominant protein pyruvate dehydrogenase complex dihydrolipoamide acetyltransferase (BMEI0856) is a part of the Pyruvate Dehydrogenase Complex, which is reported to be involved in protein synthesis during *Brucella* stress response (Teixeira-Gomes et al., 2000; Foth et al., 2005) The D-ribose-binding-periplasmic protein precursor (BMEI1390) is one of the sugar-binding proteins that help in sugar uptake to synthesize complex cell constituents (Wagner et al., 2002). The DNA starvation stationary phase protection (Dps, BMEI1980) is a part of the stress response system that binds non-specifically to DNA during oxidative stress (Martinez and Kolter, 1997; Roop 2nd et al., 2003). BMEII1048 encodes the molecular chaperonin, GroEL, a member of the heat shock protein family that plays a vital role in the structure and folding of other proteins (Abbady et al., 2012). GroEL has been reported to be a potent inducer of host immune responses (Leclerq et al., 2002). The 50S ribosomal protein L7/L12 (BMEI0748) is associated with translation initiation and is critical for ribosomal translocation (Carlson et al., 2017). The protective efficiency of this serodominant protein has been confirmed earlier, therefore L7/L12 was employed as the positive control in this study. BMEI0855 gene encodes the pyruvate dehydrogenase subunit beta of the pyruvate dehydrogenase protein complex that catalyzes the overall conversion of pyruvate to acetyl-CoA and CO_2_. The expression of this protein is reported to be down-regulated under heat stress (Teixeira-Gomes et al., 2000). BMEII0154 codes for MotB family protein that is a part of the stator of the flagellar motor protein complex. MotB mutations have been studied earlier to determine their vital role in establishing chronic infection (Fretin et al., 2005). The genes BMEI0916 and BMEI0063 code for hypothetical proteins where the functions of these proteins are yet to be established.

Our experimental data suggest that these serodominant proteins are potential candidates for developing next-generation vaccines for brucellosis. To validate this, a challenge study was performed to examine the protective efficiency of the serodominant proteins against *B. melitensis* in mice. The serodominant protein, BMEI0856 that induced a Th1 response conferred a significant level of protection against challenge with *B. melitensis*. The protective efficacy of BMEI0856 has appeared to be superior to that of the reported *Brucella* vaccine candidate, L7/L12. Even though we observed induction of Th1 type response by BMEI0063, it did not confer any significant level of protection against *B. melitensis* challenge compared to BME0856 and L7/L12. The serodominant protein, BMEI1513, that induced a Th2 type response failed to confer any protection highlighting the importance of Th1 type immune response for clearance of *Brucella*. The preliminary data obtained from the *in vivo* challenge study indicate that BMEI0856 could serve as an ideal candidate for developing a next-generation vaccine for brucellosis. Our future experiments will examine the utility of other serodominant proteins for vaccine development.

Antigens that induce a robust antibody response can serve as ideal candidates for developing improved serodiagnosis assays. We evaluated the seroreactivity of purified serodominant proteins by immunoblotting with brucellosis-positive and negative serum samples from cattle and humans. Even though all the tested proteins reacted with brucellosis-positive serum samples with different efficiency, Dps protein exhibited strong seroreactivity with both animal and human sera. We did not observe any cross-reactivity of Dps protein with serum samples from healthy animals or humans. The Dps protein is a part of Proteobacteria’s σE1 stress response system and is reported to play a crucial role in the stress survival and chronic infection of *Brucella* (Kim et al., 2013). A recent study demonstrated that *Brucella* secretes Dps protein in the infected macrophages that mediate ferritinophagy activation and host cell necrosis to facilitate the egress and bacterial dissemination (Hop et al., 2023). This may release Dps into the circulatory system to generate a potent antibody response in the *Brucella*-infected host.

A DIVA-capable serodiagnostic assay is essential when a whole-cell vaccine is used for administration. The principle of DIVA is eliciting an antibody response against the pathogen that is different from the response induced due to vaccination. Thus, distinguishing an infected animal from the vaccinated in a serological analysis is based on the antigen lacking in the vaccine candidate. DIVA vaccines may lack one or more immunological protein antigens that are present in the natural form of a pathogen. A previous study had identified many proteins, including Dps that were differentially expressed in the *B. abortus* and *B. abortus* S19 vaccine strain (Lamontagne et al., 2009). However, no experimental validation of this data was performed to examine the serodiagnostic potential of Dps protein for developing improved sero-monitoring assays with DIVA capability. Since our screening also identified Dps protein and showed robust immunogenicity in mice, we sought to examine its potential to serve as a candidate for developing improved serodiagnostic assays. Detailed immunoprobing of purified Dps protein with serum samples from naturally infected or *B. abortus* S19-vaccinated cattle indicated that Dps exhibits DIVA capability where it reacted only with the sera from naturally infected cattle and not with the serum samples from *B. abortus* S19-vaccinated animals. Further, no seroreactivity was observed when Dps protein was probed with sera from cattle after various days of *B. abortus* S19 vaccination. These data imply that the S19 vaccine strain of *B. abortus* may not express Dps protein. However, the Dps gene is present on chromosome I of *B. abortus* S19, similar to its parent strain, *B. abortus* (Pajuaba et al., 2012). Therefore, we examined whether Dps protein is expressed in the *B. abortus* S19 strain. Our subsequent immunoblotting experiments indicated a minimal expression of Dps protein in the *B. abortus* S19 strain compared to the wild-type *B. abortus*. Since the expression of Dps is low in the *B. abortus* S19 strain, it appears that the antibody response in the vaccinated animals is undetectable, which provides the DIVA capability.

Given that Dps protein reacted with brucellosis-positive serum samples from animals and humans and exhibited DIVA capability, we wished to develop Dps-based serodiagnostic assays. A prototype LFA based on the Dps protein efficiently detected brucellosis-positive serum samples from animals and humans indicating its utility in point-of-care applications. Further, the LFA could differentiate vaccinated from naturally infected cattle. Toward developing the iELISA, we optimized various assay parameters such as Dps concentration, blocking agent, and optimal serum and secondary antibody dilutions. Subsequently, we prepared the iELISA kits and compared their efficiency with the commercially available brucellosis detection kits. An in-house assay validation with human sera samples showed 99% sensitivity and 98% specificity for the Dps protein-based iELISA. Further, the Dps-based iELISA and LFA showed DIVA capability with bovine serum samples where the vaccinated cattle were assay negative compared to the naturally infected animals. The cross-reactivity with the immune sera of Gram-negative bacteria, especially *Y. enterocolitica* O:9, *S. typhimurium*, and *E. coli* is a major drawback of existing LPS-based serodiagnostic assays for brucellosis. Our experimental data indicate that Dps-based iELISA does not exhibit any cross-reactivity with the immune sera of *Y. enterocolitica* O:9 or *S. typhimurium.* Collectively, our studies suggest that Dps-based LFA and iELISA could serve as ideal diagnostic tools for brucellosis control programs where assays with high sensitivity, specificity, and DIVA capabilities are required.

## Conclusion

Our high throughput immunoprobing of *B. melitensis* protein microarray identified many potential candidates for developing improved vaccines and serodiagnostic assays for animal and human brucellosis. Some of the serodominant proteins induced a robust Th1-type response indicating their potential to serve as ideal candidates for developing next-generation vaccines. In accordance with this, BMEI0856 conferred protection against challenge with *B. melitensis* in mice. Among the identified serodominant proteins, Dps exhibited robust seroreactivity with brucellosis-positive sera from both humans and animals except with *B. abortus* S19-vaccinated cattle sera. The Dps-based LFA and iELISA exhibited high sensitivity, specificity, and DIVA capability. The recombinant Dps protein, peptides from Dps, or anti-Dps antibodies could be used for developing other sensitive point-of-care diagnostic assays and biosensors for the detection of animal and human brucellosis in a cost-effective manner.

## Data availability statement

The original contributions presented in the study are included in the article/Supplementary material, further inquiries can be directed to the corresponding author.

## Ethics statement

The studies involving serum samples from human subjects were approved and collected by ICAR-National Meat Research Institute, Hyderabad, Inida with approval from the Institutional Ethics Committee (Approval number: KAMSRC/IEC/04/2018). The studies were conducted in accordance with the local legislation and institutional requirements. The participants provided their written informed consent to participate in this study. All the animal studies were approved by the Institutional Biosafety Committee (Approval number: IBSC/2013/NIAB/0001B) and Institutional Animal Ethics Committee (Approval number: IAEC/2019/NIAB/34/GKR & IAEC/2021/NIAB/09/GKR) of NIAB. *In vivo* challenge studies with *B. melitensis* 16M strain were performed in the BSL-3 laboratory facility of UoH-NIAB (Approval number: IAEC/NIAB/2022/08/GKR). The study was conducted in accordance with the local legislation and institutional requirements.

## Author contributions

PN: Formal analysis, Investigation, Methodology, Validation, Writing – original draft, Writing – review & editing. PJ: Investigation, Methodology, Validation, Writing – review & editing. SM: Investigation, Methodology, Writing – review & editing. VM: Formal analysis, Investigation, Methodology, Validation, Writing – review & editing. DK: Investigation, Methodology, Writing – review & editing. RP: Investigation, Methodology, Writing – review & editing. SB: Resources, Writing – review & editing. GR: Conceptualization, Funding acquisition, Investigation, Resources, Supervision, Visualization, Writing – original draft, Writing – review & editing.
