# Supplementary material for: Immuno-profiling of Brucella proteins for developing improved vaccines and DIVA capable serodiagnostic assays for brucellosis
Source: Front Microbiol. 2023 Oct 4;14:1253349. doi: 10.3389/fmicb.2023.1253349 (PMC10582347; doi:10.3389/fmicb.2023.1253349)
Supplement: Supplementary file 1 [file Data_Sheet_1.pdf]

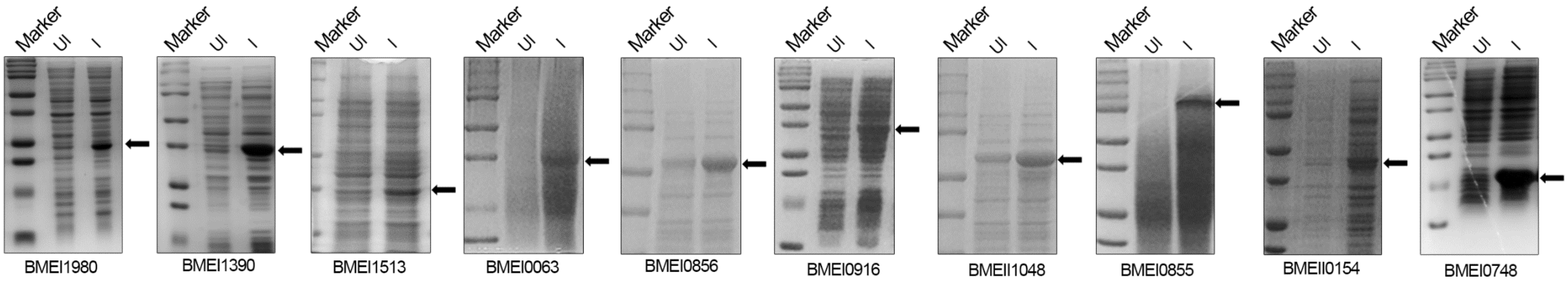

Supplementary Figure 1

The SDS-PAGE gel images showing uninduced and induced samples of BL21 *E. coli* expressing BMEI1980, BMEI1390, BMEI1513, BMEI0063, BMEI0856, BMEI0916, BMEI1048, BMEI0855, BMEII0154 and BMEI0748, respectively.

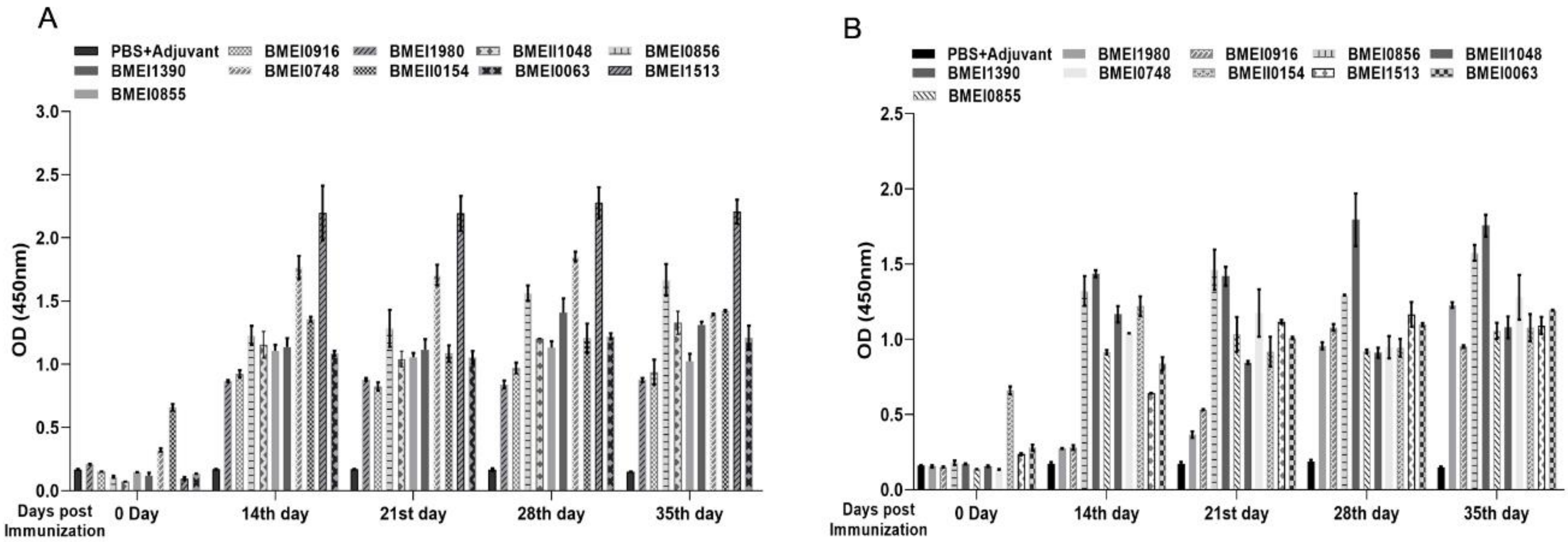

Supplementary Figure 2 (A) & (B) represent levels of IgG1 and IgG2a, respectively at the 0, 14, 21, 28 and 35 post-immunization in the serum samples. BALB/c mice were immunized with purified recombinant proteins on day 1, followed by administering a booster dose on day 21. Blood samples were collected on day 0, 14, 21, 28, 35 and 45 post-immunization. Followed by serum separation and estimation of IgG1 and IgG2a levels by ELISA.

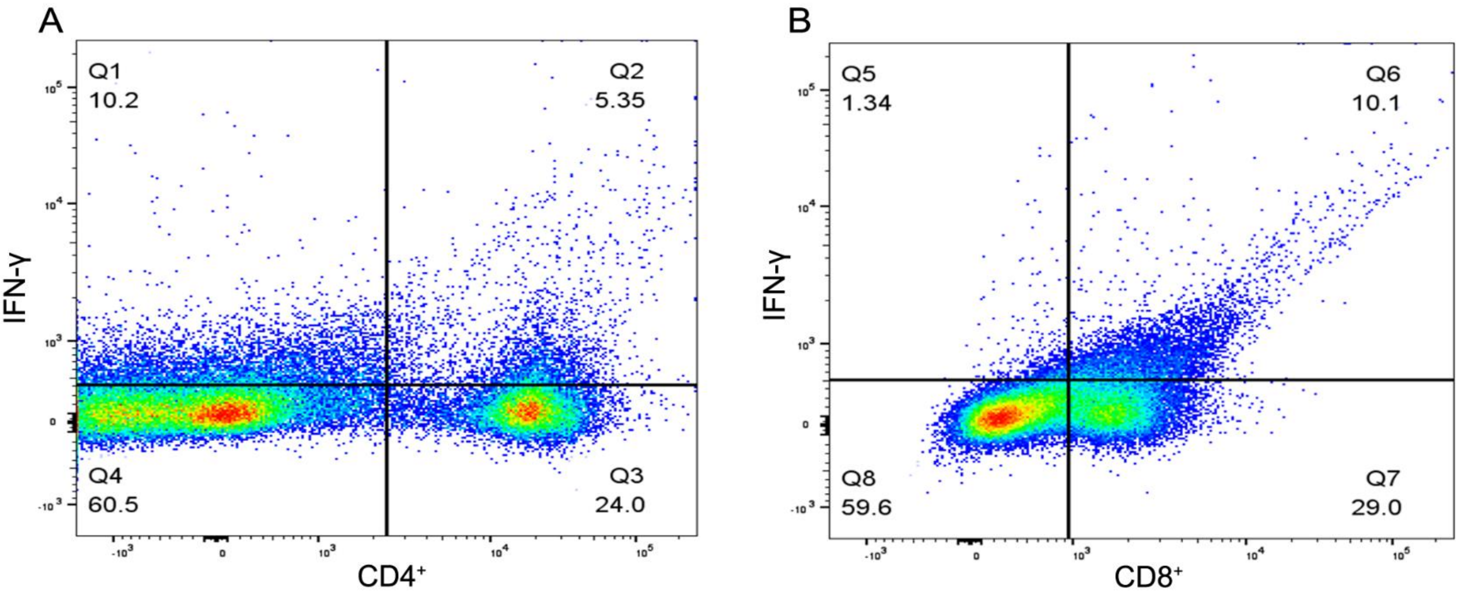

Supplementary Figure 3. (A) Representative flow cytometry plot of IFN-γ (APC-A) producing CD4$^+$ T (PE-A) cell population in mice immunized with BMEI0063. Q1& Q3 represent IFN-γ and CD4$^+$ single positive populations respectively, Q2 represents IFN-γ and CD4$^+$ double positive populations and Q4 represents IFN-γ and CD4$^+$ double negative populations. **(B)** Representative flow cytometry plot of IFN-γ (APC-A) producing CD8$^+$ T (PerCP-Cy5-A) cell population in mice immunized with BMEI0063. Q5& Q7 represent IFN-γ and CD8$^+$ single positive populations respectively, Q6 represents IFN-γ and CD8$^+$ double positive populations and Q8 represents IFN-γ and CD8$^+$ double negative populations.

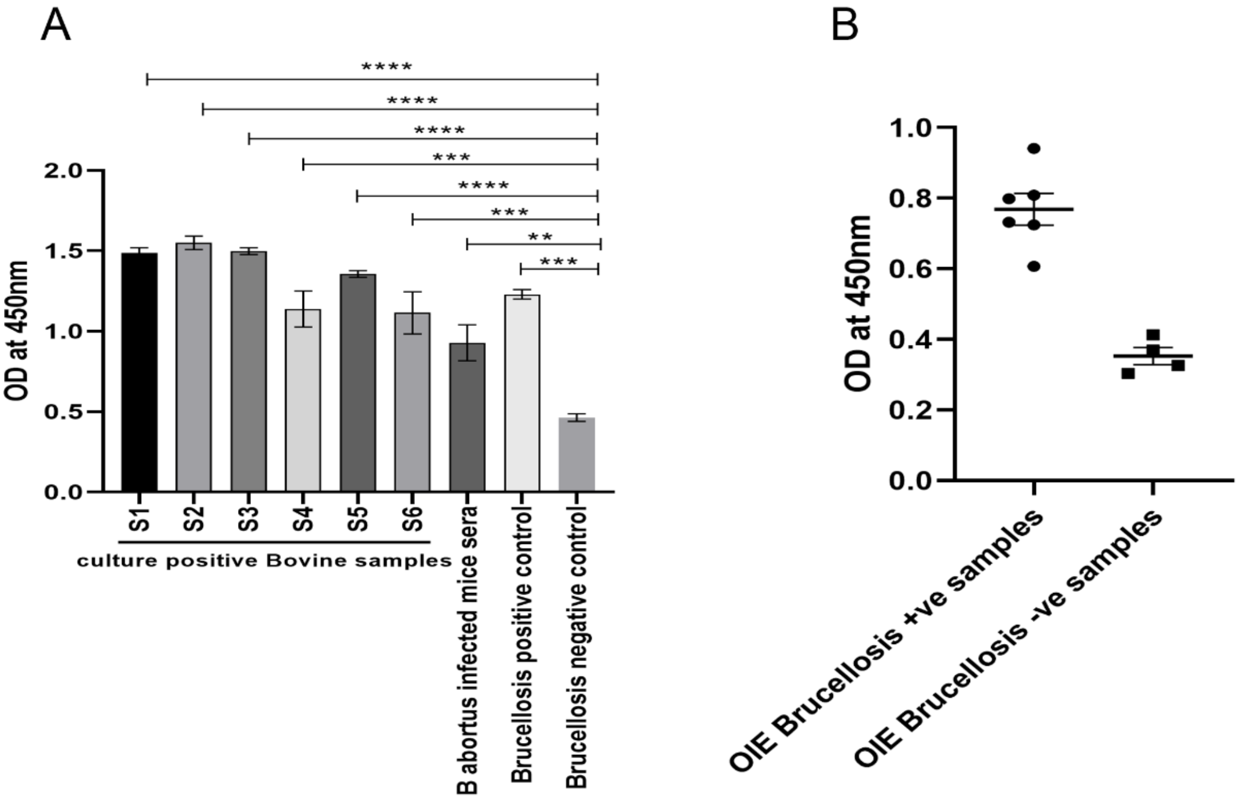

Supplementary Figure 4. (A) Screening of *Brucella* culture-positive bovine or mouse serum samples with Dps-based iELISA. Culture positive bovine or mouse serum samples were tested with Dps-based iELISA, along with commercial brucellosis positive and negative controls. The Y-axis indicate the OD values at 450 nm and X-axis shows various serum samples. **(B)** Screening of Dps-based iELISA using OIE-brucellosis positive/negative reference bovine sera. The Y-axis indicate the OD values at 450 nm and X-axis shows positive/negative serum samples.

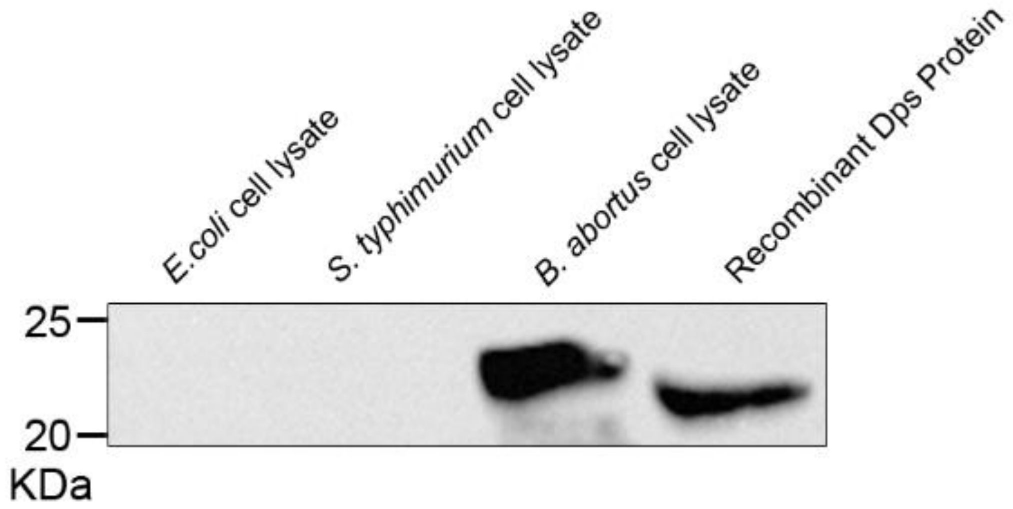

Supplementary Figure 5. Immunoblot of lysates of *E.coli*, *Salmonella Typhimurium* and *Brucella abortus* and purified recombinant Dps protein of *Brucella.* The membrane was probed with anti-Dps polyclonal antibody raised against the Dps protein of *Brucella*.
